# Hormone crosstalk in wound stress response: wound-inducible amidohydrolases can simultaneously regulate jasmonate and auxin homeostasis in *Arabidopsis thaliana*

**DOI:** 10.1093/jxb/erv521

**Published:** 2015-12-15

**Authors:** Tong Zhang, Arati N. Poudel, Jeremy B. Jewell, Naoki Kitaoka, Paul Staswick, Hideyuki Matsuura, Abraham J. Koo

**Affiliations:** ^1^Division of Biochemistry, University of Missouri, Columbia, MO 65211, USA; ^2^Interdisciplinary Plant Group, University of Missouri, Columbia, MO 65211, USA; ^3^Division of Plant Sciences, University of Missouri, Columbia, MO 65211, USA; ^4^Institute of Biological Chemistry, Washington State University, Pullman, WA 99163, USA; ^5^Laboratory of Bioorganic Chemistry, Division of Applied Bioscience, Research Faculty of Agriculture, Hokkaido University, Sapporo 060-8589, Japan; ^6^Department of Agronomy and Horticulture, University of Nebraska-Lincoln, Lincoln, NE 68521, USA

**Keywords:** Auxin, crosstalk, hormone metabolism, jasmonate, signaling, wound stress.

## Abstract

Jasmonate (JA) and auxin are essential hormones in plant development and stress responses. While the two govern distinct physiological processes, their signaling pathways interact at various levels. Recently, members of the Arabidopsis indole-3-acetic acid (IAA) amidohydrolase (IAH) family were reported to metabolize jasmonoyl-isoleucine (JA-Ile), a bioactive form of JA. Here, we characterized three IAH members, ILR1, ILL6, and IAR3, for their function in JA and IAA metabolism and signaling. Expression of all three genes in leaves was up-regulated by wounding or JA, but not by IAA. Purified recombinant proteins showed overlapping but distinct substrate specificities for diverse amino acid conjugates of JA and IAA. Perturbed patterns of the endogenous JA profile in plants overexpressing or knocked-out for the three genes were consistent with ILL6 and IAR3, but not ILR1, being the JA amidohydrolases. Increased turnover of JA-Ile in the ILL6- and IAR3-overexpressing plants created symptoms of JA deficiency whereas increased free IAA by overexpression of ILR1 and IAR3 made plants hypersensitive to exogenous IAA conjugates. Surprisingly, ILL6 overexpression rendered plants highly resistant to exogenous IAA conjugates, indicating its interference with IAA conjugate hydrolysis. Fluorescent protein-tagged IAR3 and ILL6 co-localized with the endoplasmic reticulum-localized JA-Ile 12-hydroxylase, CYP94B3. Together, these results demonstrate that in wounded leaves JA-inducible amidohydrolases contribute to regulate active IAA and JA-Ile levels, promoting auxin signaling while attenuating JA signaling. This mechanism represents an example of a metabolic-level crosstalk between the auxin and JA signaling pathways.

## Highlight

Wound-inducible and ER-located amidohydrolases with overlapping substrate specificities for IAA– and JA–amino acid conjugates regulate the production and destruction of active auxin and JA signals in wounded leaves.

## Introduction

Hormones co-ordinate developmentally programmed processes as well as responses that are induced by external stimuli. Although different hormones govern distinct biological processes, the final outcome is often the result of complex interactions among multiple hormone pathways. Deciphering the molecular mechanism of this crosstalk has been an important trend in plant hormone research (Santner and Estelle, 2009; Wasternack and Hause, 2013). This includes studies of auxin and jasmonate (JA), which carry out many indispensable functions throughout a plant’s life cycle. Auxin is necessary for the proper development of embryos, roots, and shoots, and is also well known for its role in gravitropism and phototropism (Santner and Estelle, 2009; Ludwig-Müller, 2011). JA, on the other hand, is best known for its role in plant resistance to insects and fungal pathogens (Howe and Jander, 2008; Wasternack and Hause, 2013).

Many cases of crossover between JA and auxin signaling pathways have been detected, at the level of both gene expression and metabolism (Perez and Goossens, 2013; Wasternack and Hause, 2013). For example, JA inhibits primary root growth in Arabidopsis through repressing the expression of *PLETHORA* (*PLT1*) and *PLT2*, which are key transcription factors (TFs) of the auxin-regulated root meristem specification and maintenance (Chen *et al.*, 2011). JA can promote auxin biosynthesis through transcriptional activation of *ANTHRANILATE SYNTHASE a1* (*ASA1*) and *ASB1* encoding enzymes in the l-tryptophan biosynthetic pathway, which provides precursors for auxin biosynthesis (Sun *et al.*, 2009). Transcriptional regulation of two auxin biosynthetic genes, *YUCCA8* and *YUCCA9*, is also linked to JA-induced auxin biosynthesis (Hentrich *et al.*, 2013). On the other hand, auxin can induce the expression of JA biosynthetic genes (Tiryaki and Staswick, 2002), and, in flowers, auxin-responsive TFs, AUXIN RESPONSE FACTOR 6 (ARF6) and ARF8, promote fertility through activation of JA biosynthesis (Tabata *et al.*, 2010). Auxin-inducible acyl amidosynthetases, Gretchen Hagen 3.3 (GH3.3), GH3.5, and GH3.6, participate in adventitious root initiation by modulating JA homeostasis (Gutierrez *et al.*, 2012).

Molecular perception of auxin and JA is linked to transcriptional regulation of downstream genes through 26S proteasome-dependent protein degradation mechanisms (Thines *et al.*, 2007; Santner and Estelle, 2009). The two mechanisms bear a remarkable resemblance to each other, including nuclear-residing three-component receptor complexes consisting of a hormone ligand (auxin or JA), an F-box protein, and a transcriptional repressor (Chini *et al.*, 2007; Tan *et al.*, 2007; Thines *et al.*, 2007; Perez and Goossens, 2013). In the absence of the hormones, transcription is repressed by repressor proteins, Aux/IAA (for auxin) or JASMONATE ZIM-DOMAIN (JAZ) (for JA). Rising levels of auxin or JA promote recruitment of Aux/IAA or JAZ by the SKP1/Cullin/F-box (SCF) E3 ubiquitin ligase complex, which is shared by the two hormones, except for the F-box protein which is unique for each hormone (TIR1/AFB for auxin and COI1 for JA). The ensuing events of ubiquitination and degradation of Aux/IAA or JAZ proteins unleash TFs for transcription of auxin- or JA-responsive genes. Protein interaction studies have established the strict structural requirements for the auxin and JA molecules to be able to act as ligands for binding and formation of the tertiary receptor complexes (Tan *et al.*, 2007; Thines *et al.*, 2007). For auxin, the free carboxylic acid form, indole-3-acetic acid (IAA), and its structurally related receptor agonists, and, for JA, an amino acid-conjugated form, jasmonoyl-isoleucine (JA-lle), and its close structural variants were identified as the most effective ligands. Genetic evidence supports such conclusions (Staswick and Tiryaki, 2004; Staswick *et al.*, 2005).

Because IAA and JA-Ile have direct signaling roles with global consequences, their levels must be precisely regulated. Along with biosynthesis and catabolism, conjugation to amino acids is a common way of controlling hormone levels by converting active signaling forms to inert forms for storage, transport, or degradation. Amino acid conjugates of auxin and JA occur in most plant species investigated so far (Ludwig-Müller, 2011; Wasternack and Hause, 2013). The conjugation step is catalyzed by members of the GH3 family proteins (Hagen and Guilfoyle, 1985; Staswick *et al.*, 2005). Several enzymes in Group II of the 19 Arabidopsis GH3 enzymes catalyze IAA conjugation, whereas JA-Ile is formed by JASMONATE RESISTANT1 (JAR1) in Group I of the same family (Staswick and Tiryaki, 2004; Staswick *et al.*, 2005). The reverse reaction of IAA conjugation to amino acids is catalyzed by enzymes belonging to the IAA amidohydrolase (IAH) family consisting of seven members in Arabidopsis that have previously been studied with respect to auxin metabolism (Bartel and Fink, 1995; Davies *et al.*, 1999; Ludwig-Müller, 2011). Of the seven members, IAA-LEU RESISTANT1 (ILR1), ILR1-like (ILL) 1, ILL2, and IAA-ALA RESISTANT3 (IAR3) were found to have catalytic activity towards various IAA–amino acid conjugates (LeClere *et al.*, 2002). Both *in vitro* and genetic evidence support ILR1, the founding member of the IAH family, to be an IAA-Leu hydrolase (Bartel and Fink, 1995), and IAR3, the most evolutionarily conserved member (Campanella *et al.*, 2003), to hydrolyze mainly IAA-Ala (Davies *et al.*, 1999). No *in vitro* activity against IAA conjugates was observed with ILL3, ILL5, or ILL6 (LeClere *et al.*, 2002).

Signs of connection between *IAH* genes and JA signaling were first detected with the expression of *JR3*, later reported to be identical to *IAR3* (Davies *et al.*, 1999), which was found to be induced by JA and wounding (Titarenko *et al.*, 1997). Recently, three independent groups have reported the role of IAR3 and ILL6 in JA metabolism (Woldemariam *et al.*, 2012; Bhosale *et al.*, 2013; Widemann *et al.*, 2013). The first among these studies showed that a *Nicotiana attenuata* homolog of IAR3 hydrolyzed JA-Ile *in vitro*, and that when the gene was silenced in *N. attenuata* the endogenous JA-Ile level increased (Woldemariam *et al.*, 2012). The *ILL6* gene was picked up as the top candidate regulator of the JA pathway by a novel gene expression network analysis (Bhosale *et al.*, 2013). The knock-out mutants of *ill6* displayed decreased capacity to release isoleucine from exogenously applied radioisotope-labeled JA-Ile and increased endogenous JA-Ile levels in wounded leaves, consistent with the role of ILL6 as a JA-Ile hydrolase. Recombinant IAR3 and ILL6 proteins expressed in bacteria were able to hydrolyze JA-Ile; IAR3 additionally hydrolyzed 12-hydroxy-JA-Ile (12OH-JA-Ile) (Widemann *et al.*, 2013), a major JA metabolite formed by oxidative JA-Ile catabolism (Koo and Howe, 2012). Consistently, 12OH-JA-Ile levels were increased in both *iar3* and *ill6* mutants (Widemann *et al.*, 2013; Koo *et al.*, 2014).

Here we describe the further characterization of ILL6, IAR3, and a third JA-inducible IAH family member, ILR1, for their function in JA and auxin metabolism and signaling. Catalytic activities of recombinant proteins were tested for a variety of different JA and IAA conjugates. ILR1, ILL6, and IAR3 were overexpressed in Arabidopsis and the resulting plants were analyzed for their endogenous hormone profile and for phenotypes associated with JA and auxin signaling. Higher order mutants were created to examine functional redundancies between IAHs. Subcellular localization was determined for ILL6 and IAR3 proteins. These results support a hormone crosstalk model where wound-inducible amidohydrolases simultaneously regulate the levels of JA and auxin to co-ordinate stress responses.

## Materials and methods

### Plant material, growth conditions, and treatments

*Arabidopsis thaliana* ecotype Col-0 was used as the wild type (WT) for all experiments, except for Fig. 2D–F where Wassilewskija (WS) was used instead. The *ill6-2iar3-5* double mutant was made by a genetic crossing between the T-DNA insertion lines CS852193 (*ill6-2*) (Bhosale *et al.*, 2013) and SALK_069047 (*iar3-5*) obtained from the Arabidopsis Biological Resource Center. *ilr1-1*, *ilr1-5* (Rampey *et al.*, 2006), *iar3-2*, *iar3-2ilr1-1* (Davies *et al.*, 1999), and *ILR1-OE* (Davies *et al.*, 1999) seeds were kindly provided by Dr Bonnie Bartel (Rice University). A complete list of oligonucleotide primers used for genotyping is given in Supplementary Table S2 available at *JXB* online. Plants were grown in environmental growth chambers maintained at 22 °C with a photoperiod of 16h light (100–120 μE m^−2^ s^−1^). Hormone treatments for gene expression studies were carried out either by spraying an indicated amount of hormones evenly onto the surface of fully expanded leaves of soil-grown plants or by growing seedlings on plates containing the hormone. Wounding was conducted on fully expanded rosette leaves of 4- to 5-week-old soil-grown plants by crushing across the midrib twice using a hemostat with serrated tips (Herde *et al.*, 2013).

### Chemicals

(±)-Jasmonic acid, methyl jasmonic acid (MeJA), coronatine, IAA, IAA-Ala, and d_5_-IAA were purchased from Sigma-Aldrich. JA–amino acid conjugates, 12OH-JA, 12OH-JA-Ile, 12COOH-JA-Ile, 12-*O*-Glc-JA, and 12-*O*-Glc-JA-Ile, were synthesized according to protocols described previously (Kramell *et al.*, 1988; Staswick and Tiryaki, 2004; Chung *et al.*, 2008; Koo *et al.*, 2009; Kitaoka *et al.*, 2014). IAA-Leu was synthesized according to methods described for JA conjugates (Staswick and Tiryaki, 2004) except that tetrahydrofuran was replaced with acetonitrile as the solvent.

12-Sulfonyl-JA-Ile sodium salt (12-*O*-SO_3_Na-JA-Ile) was synthesized as follows. To a stirred mixture of 12OH-JA-Ile methyl ester (29mg, 0.085 mmol) in dry pyridine (6ml), a solution of sulfur trioxide pyridine complex (135mg, 0.85 mmol) in dry pyridine (6ml) was added and stirred for 2h at room temperature. A 45ml aliquot of methanol:water (2:1) was added and the mixture was neutralized by 1M KOH. The volatile component of the reaction mixture was removed under reduced pressure to give an oil, which was purified using Si gel column chromatography (30g, chloroform:methanol:acetic acid 80:20:0.1) to produce 12-sulfonyl JA-Ile methyl ester [27mg, 0.062 mmol (2ml), 73%]. 12-Sulfonyl JA-Ile methyl ester (27mg, 0.062 mmol) in ethanol (2ml) was stirred with an aqueous solution of 1M NaOH (0.7ml) for 2h at room temperature. Following neutralization with Amberlite IR-120, the volatile component of the reaction mixture was removed under reduced pressure to give an oil, which was purified using Si gel column chromatography (20g, methanol:chloroform:acetic acid 7:3:0.1) to produce 12-sulfonyl-JA-Ile sodium salt (12-*O*-SO_3_Na-JA-Ile, 25mg, 0.012 mmol, 92%). Physical data for 12-*O*-SO_3_Na-JA-Ile are as follows. Electrospray ionization (ESI)-MS *m/z* (rel. int., %) 419 (15), 418 (100, [M-Na]^−^), 344 (22). High-resolution (HR)-ESI-MS: *m/z* 418.1544 [M-Na]^−^ (calcd. for C_18_H_28_NO_6_S; 418.1541). ^1^H-Nuclear magnetic resonance (NMR) (270 MHz, D_2_O) δ: 5.37 (2H, m), 4.15 (1H, dd, *J*=6.6, 9.9 Hz), 3.90 (2H, t, *J*=6.4 Hz), 2.49 (1H, m), 2.36-1.73 (10H, m), 1.12 (1H, m), 0.81 (3H, d, *J*=6.9 Hz), 0.75 (3H, t, *J*=7.3 Hz).

### Transgenic plants overexpressing IAR3 and ILL6

Construction of binary vector constructs for *in planta* overexpression of *ILL6* (At1g44350) (*ILL6-OE*) and *IAR3* (At1g51760) (*IAR3-OE*) was carried out by amplifying the full-length open reading frame (ORF) of each gene by reverse transcription–PCR (RT–PCR) from a total RNA from wounded WT leaves using the primer pairs indicated in Supplementary Table S2 at *JXB* online. The resulting PCR fragments were cloned into a pBI121-derived pBITS vector (Koo *et al.*, 2006) using restriction enzyme sites *Xba*I and *Xho*I for *ILL6* or *Xba*I and *Bam*HI for *IAR3*. The resulting constructs with the respective genes placed behind the *Cauliﬂower mosaic virus* (CaMV) 35S promoter (*35S:ILL6* or *35S:IAR3*) were transformed into the C58C1 strain of *Agrobacterium tumefaciens*. *Agrobacterium tumefaciens* harboring each construct was transformed into Arabidopsis using a floral dip method. Seeds harvested from the resulting plants (T_1_) were screened for resistance to kanamycin (50 μg ml^−1^). A total of 55 and 69 seedlings each from *35S:ILL6* and *35S:IAR3* that survived the kanamycin selection were tested for transgene expression by quantitative (q)RT–PCR and for JA levels. Two selected lines—one each from *ILL6-OE* and *IAR3-OE*—were further propagated eventually to obtain T_3_ homozygous lines.

### Subcellular localization studies

Vector constructs were made for each of the two proteins, IAR3 and ILL6, with cyan fluorescent protein (CFP) fused to either their N- or C-terminus. ORFs of IAR3 and ILL6 with fused CFP sequence were amplified using an overlapping PCR method with the primers indicated in Supplementary Table S2 at *JXB* online. The N-terminal fusions had CFP inserted behind the putative 23 and 24 amino acid signal peptide sequences of IAR3 and ILL6, respectively. The C-terminal fusion for IAR3–CFP preserved the IAR3’s ER retention ‘KDEL’ motif at the very end, whereas ILL6–CFP did not have any ER retrieval sequence at the end. The amplified fragments were cloned into a Gateway binary expression vector, pGWB2 (Nakagawa *et al.*, 2007). The resulting constructs were transiently expressed in *Nicotiana benthamiana* leaves by syringe infiltration of the *Agrobacterium* C58C1 strains harboring each construct (Koo *et al.*, 2009). A second strain of *Agrobacterium* containing a *CYP94B3-mRFP* construct with previously demonstrated ER localization (Koo *et al.*, 2014) was co-infiltrated. After 48h of infiltration, fluorescent images were acquired using a Leica TCP SP8 confocal microscope.

### Recombinant proteins and *in vitro* hydrolysis assays

ORFs of ILL6 and IAR3 lacking 24 and 23 amino acids, respectively, of predicted N-terminal signaling sequences were PCR amplified (Supplementary Table S2 at *JXB* online) and cloned in between the *Eco*RI and *Sal*I sites of the pGEX-6P-1 vector (GE Healthcare) to obtain *pGEX-ILL6* and *pGEX-IAR3*, which placed a glutathione *S*-transferase (GST) tag on the N-terminus of ILL6 or IAR3. Sequence-verified *pGEX-ILL6* and *pGEX-IAR3* were each transformed into *Escherichia coli* strain BL21 (DE3). The *pGEX-ILR1* plasmid was obtained from Dr Bonnie Bartel (Rice University) (LeClere *et al.*, 2002), and was transformed into the same BL21 (DE3) strain. Protein expression was induced at mid-log phase by adding 0.5mM isopropyl-β-d-1-thiogalactopyranoside (IPTG) and culturing for another 16–20h at 16 °C. GST·Bind™ Kits (Novagen) were used to purify GST-fused proteins following the manufacturer’s instructions. The purity of recombinant proteins estimated by SDS–PAGE was >90%.

A previously described *in vitro* hydrolysis assay procedure (LeClere *et al.*, 2002) was adopted to test enzymatic activities of the purified GST–ILL6, GST–IAR3, and GST–ILR1 proteins. A typical reaction mixture consisted of 50mM TRIS-HCl (pH 8.0), 1mM MnCl_2_, 1mM dithiothreitol, 5 μg of puriﬁed protein, and the indicated amounts of substrates in a 25 μl reaction volume. GST proteins purified from an *E. coli* strain transformed with an empty pGEX-6P-1 vector were used as a control. The reaction was carried out at 28 °C for the indicated times and was terminated by adding 75 μl of stop solution consisting of 70% aqueous methanol and 0.5% acetic acid spiked with 0.25 μM dihydro-jasmonic acid (dhJA) and 0.5 μM d_5_-IAA as internal standards. A 5 μl aliquot of the centrifugation-cleared supernatant was directly injected for LC-MS.

### RNA analysis

RNA was extracted using TRIzol reagent (Invitrogen) from 50–100mg of frozen tissue ground into a fine powder. First-strand cDNA was synthesized from 1 μg of total RNA treated with DNase I (Qiagen) using oligo(dT)_20_ primers and Moloney murine leukemia virus (M-MLV) reverse transcriptase (Promega). A 5ng aliquot of the resulting cDNA was used as template for subsequent PCR steps. Phusion *Taq* polymerase (Thermo Scientific) and Taq-Pro Red Complete (Denville) were used as DNA polymerases for cloning purposes and for semi-quantitative RT–PCR experiments, respectively. qRT–PCR was performed on a CFX96 Touch™ real-time PCR detection system (Bio-Rad) using SsoFast™ EvaGreen^®^ Supermix (Bio-Rad) as per the manufacturer’s instructions. Relative transcript abundance was calculated after normalization with *ACTIN8* (AT1G49240) as an internal reference gene. For time course experiments, fold change compared with the 0h time point of the WT was plotted. All other graphs show transcript levels relative to *ACTIN8*.

### Metabolite analysis by mass spectrometry

Hormone metabolites were quantified using ultra-performance liquid chromatography–tandem MS (UPLC-MS/MS) (Acquity/Xevo TQ-S system, Waters) based on methods described previously (Koo *et al.*, 2014). Characteristic MS transitions were monitored using multiple reaction monitoring in ESI positive mode for IAA (*m/z*, 176>130), d_5_-IAA (181>135), and ESI negative mode for JA (*m/z*, 209→59), dhJA (211→59), 12OH-JA (225→59), 12COOH-JA (239→59), 12-HSO_4_-JA (305→97), 12-*O*-Glc-JA (387→207), JA-Ile (322→130), [^13^C_6_]JA-Ile (328→136), 12OH-JA-Ile (338→130), 12COOH-JA-Ile (352→130), 12-HSO_4_-JA-Ile (418→130), and 12-*O*-Glc-JA-Ile (500→130). Data analysis was carried out using MassLynx 4.1 and TargetLynx software (Waters).

## Results

### Transcripts of *ILR1*, *ILL6*, and *IAR3* are induced by JA or wounding but not by IAA

Earlier gene expression studies have identified members of the *IAH* gene family as being induced by wounding or JA treatment (Titarenko *et al.*, 1997; Bhosale *et al.*, 2013; Widemann *et al.*, 2013). To gain a more comprehensive understanding of the JA-inducible nature of the *IAH* transcripts, we conducted a time course qRT–PCR analysis of all seven *IAH* genes (Supplementary Fig. S1 at *JXB* online). Mature rosette leaves of 4-week-old Arabidopsis plants were either mechanically wounded or sprayed with a 100 μM MeJA solution, and sampled at 0, 0.5, 1, 4, 8, and 12h after treatment. Transcripts of three genes, *IAR3*, *ILL6*, and *ILR1*, were strongly induced by MeJA. The induction was transient, rising within a few hours of treatment and peaking at 4h, following typical ‘early gene’ kinetics (Chung *et al.*, 2008). Wounding also induced expression of *IAR3*, *ILL6*, and *ILR1*, and, additionally, *ILL5*, which is a pseudogene in the Col-0 ecotype (LeClere *et al.*, 2002; Widemann *et al.*, 2013). No transcript increase by either treatment was detected in *coi1-1* (Supplementary Fig. S1B, D), indicating a strict requirement for the functional JA perception pathway. The promoter regions of all three genes contained canonical MYC2-binding *cis*-elements (or G-box) according to the Arabidopsis Gene Regulatory Information Server (http://arabidopsis.med.ohio-state.edu/), and earlier microarray data indicated that their expression is regulated by MYC2 and MYC3 (Dombrecht *et al.*, 2007; Cheng *et al.*, 2011; Nakata *et al.*, 2013).

We next tested if their expression can be induced by IAA treatment (Supplementary Fig. S1E at *JXB* online). Unlike the two IAA-responsive marker genes, *IAA5* and *GH3.1*, which were strongly induced by IAA, none of the *IAH* transcripts responded to IAA treatment. This is in contrast to the *GH* family genes encoding the IAA–amino acid-conjugating enzymes, which were strongly induced by IAA (Hagen and Guilfoyle, 1985; Staswick *et al.*, 2005), showing that although IAH enzymes function in auxin metabolism, their gene expression is not controlled by IAA, but rather by JA.

### Substrate specificities of recombinant ILR1, IAR3, and ILL6 proteins

To test their catalytic activities, ILR1, IAR3, and ILL6 were expressed in *E. coli* as N-terminal GST fusions. Conditions for the *in vitro* hydrolysis assay were first determined by incubating purified recombinant proteins with varying concentrations of JA-Ile substrate for different times, and measuring the amount of released free JA (Supplementary Fig. S2 at *JXB* online). A linear increase of JA was maintained for at least 3h for both GST–IAR3 and GST–ILL6 (Supplementary Fig. S2A) under the chosen assay conditions (see the Materials and methods). GST–IAR3 displayed at least several times higher hydrolytic activity compared with GST–ILL6 under all tested conditions, while purified GST protein alone showed no significant hydrolytic activity (Table 1). GST–ILR1 did not show any detectable activity against JA-Ile substrate even at relatively high substrate concentrations, even though the enzyme was highly active against the IAA-Leu substrate (Supplementary Fig. S2B, C). Next, the three proteins were tested against 10 different conjugates of JA (Table 1). Among the 10 were six with different amino acids (-Ile, -Leu, -Val, -Gln, -Thr, -Phe) and four with modification at the C_12_ position of JA-Ile (12OH-, 12COOH-, 12-*O*-Glc-, 12-HSO_4_-). GST–IAR3 hydrolyzed all six amino acid-substituted conjugates comparably well. Although hydroxylation at C_12_ had no effect on GST–IAR3 activity, further modifications to 12COOH-, 12-*O*-Glc-, or 12-HSO_4_- almost completely eliminated the cleavage activity.

**Table 1.** Substrate specificity of recombinant amidohydrolase enzymes

Substrate	GST–IAR3	GST–ILL6	GST–ILR1	GST
JA-Ile	3563.6±110.5^*a*^	512.3±1.4	25.3±1.1	0.4±0.4
JA-Leu	5066.8±213.9	236.9±1.0	881.8±138.2	5.2±1.9
JA-Val	3093.1±107.5	87.7±2.7	107.5±6.5	2.4±0.9
JA-Gln	2672.7±169.1	80.8±4.1	158.0±7.0	23.0±1.1
JA-Thr	4016.0±155.9	132.3±3.5	36.0±1.7	10.7±1.1
JA-Phe	3812.7±67.0	76.5±6.4	1798.0±58.9	21.8±3.8
12OH-JA-Ile	2672.7±646.1	128.6±17.7	59.8±1.6	72.8±2.4
12COOH-JA-Ile	207.7±4.3	3.5±0.0	7.8±0.2	7.9±0.4
12-*O*-Glc-JA-Ile	47.1±4.2	4.3±1.3	5.2±6.6	6.7±6.1
12-HSO_4_-JA-Ile	277.9±26.0	3.0±0.4	1.4±1.5	1.1±0.8
IAA-Ala^*b*^	27515.5±438.7	25.4±4.1	284.0±16.7	19.9±1.8
IAA-Leu	78.9±3.4	11.7±4.0	2581.0±78.7	8.6±1.9

^*a*^ Specific activity (pmol h^−1^ mg^−1^ of protein) was determined by quantifying JA, 12OH-JA, 12COOH-JA, 12-*O*-Glc-JA, 12-HSO_4_-JA, and IAA after 2h incubation of 5 μg of purified proteins with 4 μM of the indicated conjugate substrates. Each value represents the mean ±SD of three replicates.

^*b*^ For IAA conjugates, total reaction time was 0.5h.

GST–ILL6 displayed the highest activity toward JA-Ile even though its activity was an order of magnitude lower than that of GST–IAR3. Its relative activity also varied more widely between substrates compared with GST–IAR3. For example, its activity decreased 4- to 6-fold when the -Val, -Gln, and -Phe conjugates of JA were used instead of JA-Ile. GST–ILL6 was able to hydrolyze 12OH-JA-Ile, but, as with GST–IAR3, further modifications at the C_12_ position eliminated the activity. GST–ILL6 showed essentially no activity against IAA-Ala or IAA-Leu, making ILL6 a specialized enzyme for JA conjugates. Even though GST–ILR1 did not show any activity for JA-Ile, it was active against JA-Leu and JA-Phe. This stark difference in preference for JA-Leu over JA-Ile was somewhat surprising, but a similar strict preference of ILR1 for -Leu over -Ile conjugates was observed with the IAA conjugates (Bartel and Fink, 1995).

Overall, these *in vitro* studies show that IAR3 has broad substrate specificity for conjugates of both JA and IAA, whereas ILL6 is more specific for JA conjugates. Even though ILR1 also showed dual substrate specificity for IAA and JA conjugates, its lack of activity against major JA conjugates, i.e. JA-Ile and 12OH-JA-Ile, predicts its limited role in JA homeostasis.

### In vivo function of ILR1, IAR3, and ILL6 in JA metabolism

*In planta* overexpression was employed to study the enzymatic function of ILL6, IAR3, and ILR1 *in vivo*. Stably transformed lines of Arabidopsis plants expressing the *ILL6* or *IAR3* genes under the control of the constitutive CaMV35S promoter, designated as *ILL6-OE* and *IAR3-OE*, respectively, were generated (Supplementary Figs S3, S4 at *JXB* online). Among 55 T_1_ lines of *ILL6-OE* resistant to antibiotic selection, eight lines (lines 5, 19, 23, 42, 46, 47, 63, and 69) had markedly increased *ILL6* transcripts. The same eight lines were also severely reduced in JA-Ile content, consistent with increased JA-Ile hydrolysis by ILL6 overexpression. *IAR3-OE* lines also displayed strong correlation between the increased *IAR3* transcripts and reduced JA-Ile levels, and were established following a similar procedure to *ILL6-OE* (Supplementary Fig. S4). Two lines each from *ILL6-OE* (lines 5 and 47) and *IAR3-OE* (lines 4 and 42) were further propagated to generate T_3_ homozygous lines. Reduced JA-Ile levels were maintained in these homozygous lines, with levels in each representative line comparable with one another for both *ILL6-OE* and *IAR3-OE* progeny (Supplementary Fig. S5).

**Fig. 1.**
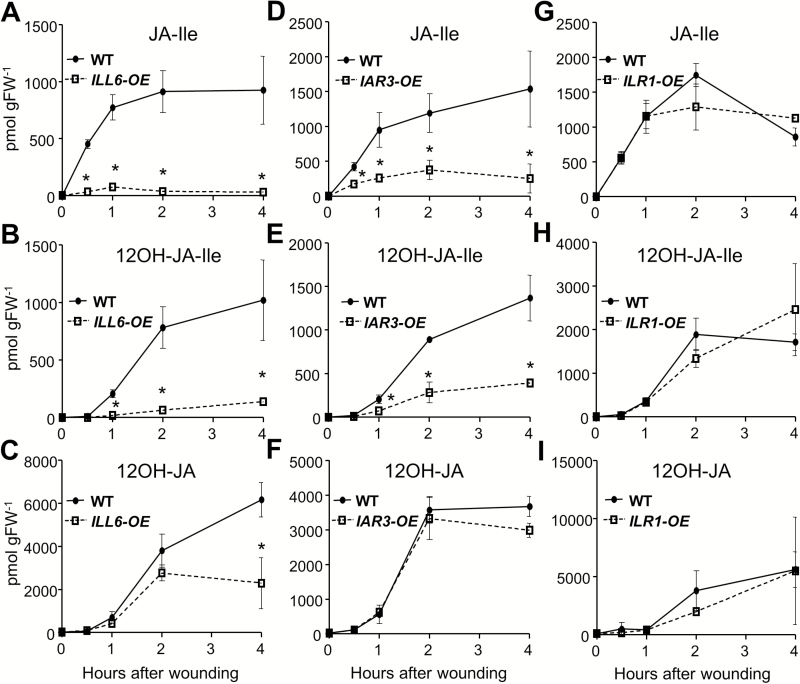
Time course of JA metabolite accumulation in wounded leaves of the WT, *ILL6-OE*, *IAR3-OE*, and *ILR1-OE*. Mechanically damaged leaves at the indicated times after wounding were analyzed for JA-Ile (A, D, G), 12OH-JA-Ile (B, E, H), and 12OH-JA (C, F, I) contents using UPLC-MS/MS. Asterisks denote a significant difference at *P*<0.05; Student’s *t*-test. Each data point represents the mean ±SD of three biological replicates.

**Fig. 2.**
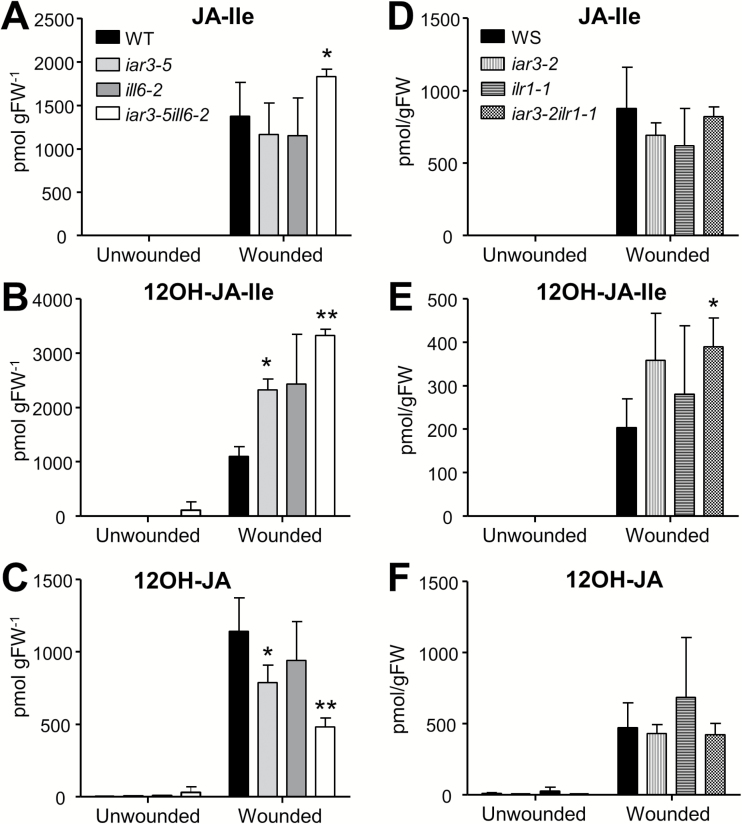
Loss-of-function mutants of IAR3 and ILL6, but not ILR1, hyperaccumulate JA-Ile and 12OH-JA-Ile. Endogenous JA-Ile (A, D), 12OH-JA-Ile (B, E), and 12OH-JA (C, F) contents were quantified from wounded (2h) and unwounded leaves of 4-week-old plants. Asterisks denote a significant difference at *P*<0.05 compared with the WT (*) or *iar3-5* (**); Student’s *t*-test. Each data point represents the mean and SD of three biological replicates.

A detailed time course JA profiling was carried out on one each of these homozygous *ILL6-OE* (line 5) and *IAR3-OE* (line 42) lines, along with a previously reported *ILR1-OE* line (Davies *et al.*, 1999) (Fig. 1). JA-Ile was strongly reduced in both *ILL6-OE* and *IAR3-OE* plants throughout the time course (Fig. 1A, D). *ILL6-OE* performed surprisingly well considering the lower hydrolytic activity (10 times less than GST–IAR3) that GST–ILL6 had shown in the *in vitro* assays (Table 1). In addition to JA-Ile, there was a major reduction in the 12OH-JA-Ile level in both *ILL6-OE* and *IAR3-OE* (Fig. 2B, E, H), which was consistent with their *in vitro* enzyme assay results. 12OH-JA, the product of 12OH-JA-Ile hydrolysis, in both *ILL6-OE* and *IAR3-OE* was similar to (before 2h) or lower than (4h) the level in the WT (Fig. 1C, F). The increased rate of 12OH-JA-Ile hydrolysis and decreased pool size of the precursor (12OH-JA-Ile) were likely to have contributed to this mild decrease in 12OH-JA. Similarly, no significant changes were observed in the JA level in these plants (Supplementary Fig. S6 at *JXB* online). *ILR1-OE* did not show any change in the major JA metabolite profile, consistent with the *in vitro* assay results.

**Fig. 3.**
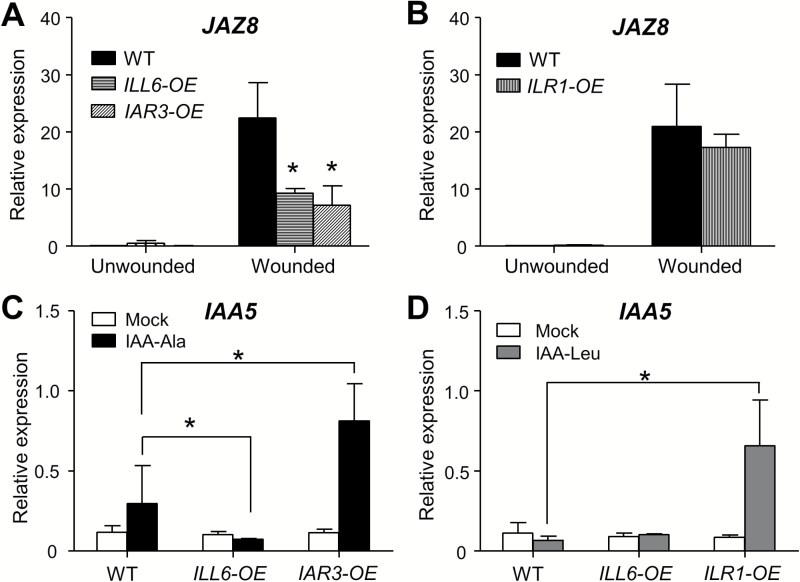
Overexpression of amidohydrolases impacts JA and IAA marker gene expression. (A and B) qRT–PCR analysis of *JAZ8* expression in unwounded and wounded (2h) leaves of the WT, *ILL6-OE* (line 5), *IAR3-OE* (line 42), and *ILR1-OE*. Fold change relative to the unwounded WT transcript level is displayed. (C and D) *IAA5* expression in 9-day-old seedlings grown on MS medium containing the mock treatment, 50 μM IAA-Ala, or 30 μM IAA-Leu. Expression levels relative to *ACTIN8* are displayed. Error bars denote the SD of three biological replicates. Asterisks indicate a significant difference at *P*<0.05 compared with the WT; Student’s *t*-test.

Next, we analyzed loss-of-function mutants to test if the endogenous JA profiles would show opposite trends to the overexpressors (Fig. 2). To combat gene redundancy problems, double homozygous mutants, *iar3-5ill6-2* (Supplementary Fig. S7 at *JXB* online) and *iar3-2ilr1-1* (Davies *et al.*, 1999), were analyzed along with the single mutants. JA-Ile contents in wounded leaves of the single mutants, *iar3-5* and *ill6-2*, were not different from the WT, as noted in earlier reports (Widemann *et al.*, 2013; Koo *et al.*, 2014), but the levels were significantly higher (*P*<0.05) in the *iar3-5ill6-2* double mutant compared with the WT or the two single mutants, indicating that the JA-Ile turnover was strained in this double mutant (Fig. 2A). The impact on 12OH-JA-Ile was even greater, displaying close to 2-fold increases in the single mutants and up to 3-fold increases in the double mutant compared with the WT levels (Fig. 2B). The trend was reversed with 12OH-JA; that is, reduced in each single mutant and still more reduced in the double mutant (Fig. 3C). On the other hand, the JA profile in *iar3-2ilr1-1* was not significantly different from that of *iar3-2*, indicating that there was no additive contribution by *ilr1-1* mutation (Figs. 3D , F).

Together, these results show that ILL6 and IAR3 act redundantly *in vivo* to metabolize JA-Ile and 12OH-JA-Ile, whereas ILR1 has a limited role in overall JA metabolism. A previous study (Rampey *et al.*, 2004) revealed elevated levels of IAA-Ala and IAA-Leu in a *ilr1iar3ill2* triple mutant, establishing the *in vivo* function of ILR1 and IAR3 in hydrolyzing endogenous IAA–amino acid conjugates.

### IAH overexpression oppositely impacts expression of JA- and IAA-responsive genes

Constitutive activation of the JA-Ile turnover pathway in *ILL6-OE* and *IAR3-OE* is expected to have negative signaling consequences on JA-Ile-regulated gene expression. On the other hand, liberation of IAA from the amino acid conjugates by overexpression of *IAR3* and *ILR1* is expected to activate IAA-responsive gene expression. We tested this hypothesis in *IAR3-OE*, *ILL6-OE*, and *ILR1-OE* plants using a JA-Ile-responsive and an IAA-responsive marker gene, *JAZ8* (Shyu *et al.*, 2012) and *IAA5* (Dharmasiri *et al.*, 2003), respectively. *JAZ8* transcripts increased >20-fold by wounding in WT leaves (Fig. 3A, B). *JAZ8* transcripts in *IAR3-OE* and *ILL6-OE* also increased upon wounding, but the induction was less than half that in the WT (Fig. 3A). This is correlated with the strongly depleted JA-Ile in these lines (Fig. 1A, D). Consistent with the unaltered JA-Ile levels in *ILR1-OE* (Fig. 1G), *JAZ8* gene expression levels were not changed compared with the WT in *ILR1-OE* (Fig. 3B). To test auxin response, the WT and the overexpressing lines were grown on Murashige and Skoog (MS) medium supplemented with IAA-Ala or IAA-Leu, or mock solutions. *IAA5* transcript levels increased in both the IAA-Ala treated WT and *IAR3-OE* plants compared with mock treatment, but the increase was much greater in *IAR3-OE* (Fig. 3C), consistent with the explanation that a higher amount of IAA is generated by the constitutive hydrolysis of IAA-Ala in *IAR3-OE*. Similarly, *IAA5* transcripts were strongly induced in *ILR1-OE* grown on IAA-Leu plates, consistent with ILR1’s enzymatic function as an IAA-Leu hydrolase (Fig. 3D). *IAA5* gene expression was not induced in *ILL6-OE* by either IAA-Ala or IAA-Leu (Fig. 3C, D), consistent with the lack of *in vitro* hydrolytic activity of ILL6 against IAA conjugate substrates (Table 1). Similar results were obtained using *JAR1* and *GH3.1* as additional marker genes, corroborating the above observations (Supplementary Fig. S8 at *JXB* online). These gene expression results show that JA-Ile hydrolysis by IAR3 and ILL6 can attenuate JA-responsive gene expression and, at the same time, IAA-Ala and IAA-Leu hydrolysis by IAR3 and ILR1 can activate IAA-responsive gene expression in plants.

### ILL6-OE and IAR3-OE display JA-deficient phenotypes

**Fig. 4.**
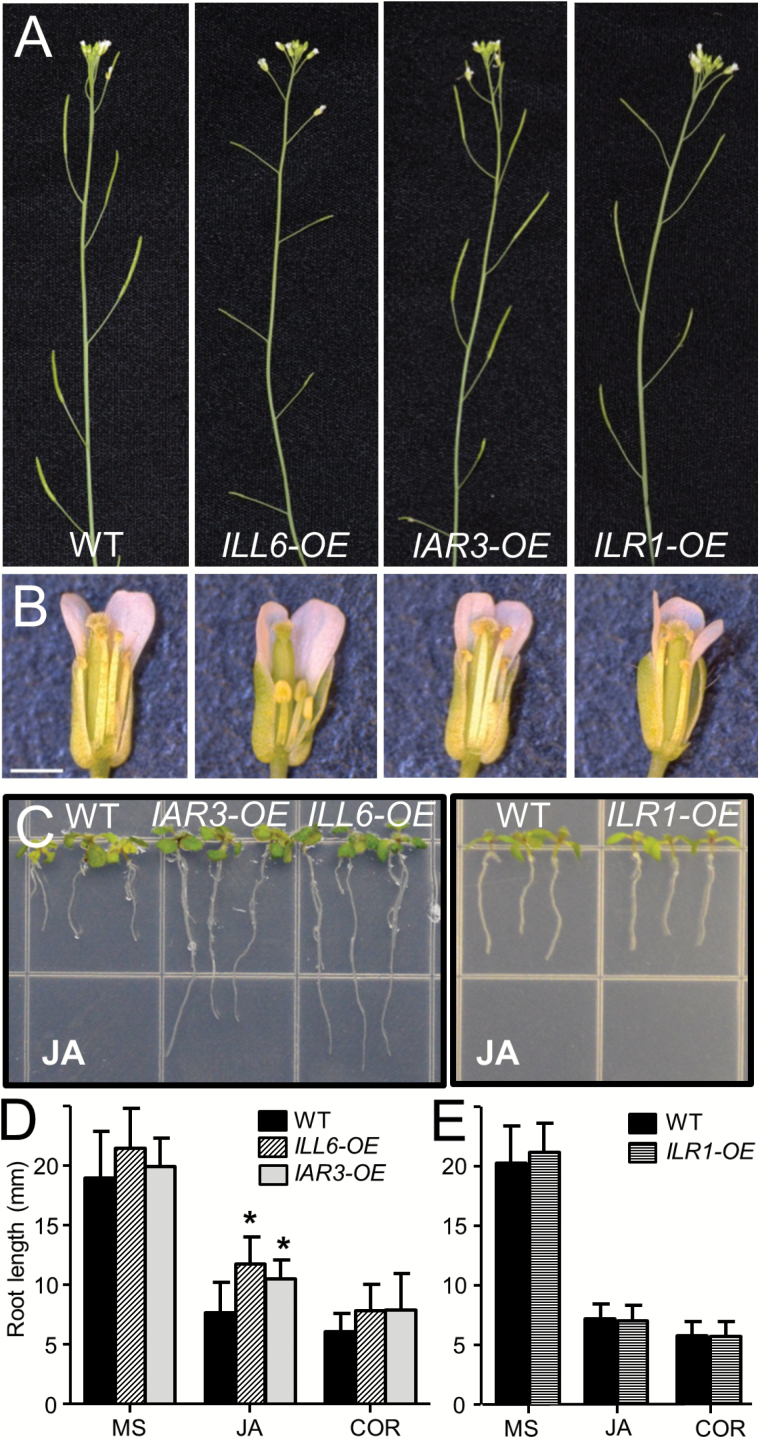
*ILL6-OE* and *IAR3-OE* plants display JA-deficient phenotypes. (A and B) Photographs of representative stems and flowers of the WT, *ILL6-OE* (line 5), *IAR3-OE* (line 42), and *ILR1-OE*. Underdeveloped siliques and short anther filaments are observed for *ILL6-OE*. Scale bar=1mm. (C–E) Photo images and root length measurements of 9-day-old seedlings grown vertically on MS medium supplemented with 20 μM jasmonic acid (C–E) or 0.5 μM coronatine (COR) (D and E). Data show the mean and SD (*n* >18). Asterisks denote a significant difference compared with the WT at *P*<0.05; Student’s *t*-test. (This figure is available in colour at *JXB* online.)

We next investigated whether biochemical and molecular changes caused by ectopic expression of IAHs result in any visible plant symptoms. Among the eight *ILL6-OE* lines selected from the T_1_ screening (Supplementary Fig. S3 at *JXB* online), two independent lines (lines 5 and 47) displayed partial fertility defects, which were heritable in the following generations (Fig. 4A, B). Over half of the siliques in these plants were underdeveloped, with few or no seeds inside (Supplementary Table S1). Developing flowers of these plants exhibited short anther ﬁlaments and delayed dehiscence (Fig. 4B), which are typical symptoms of JA-Ile deficiency in Arabidopsis (Browse, 2009). Increased JA-Ile turnover by overexpression of CYP94B enzymes created similar reproductive defects (Koo *et al*., 2011, 2014). Auxin also plays a role in flower development through JA, but JA biosynthesis is downstream of auxin signaling (Nagpal *et al.*, 2005). Unlike *ILL6-OE*, no apparent fertility defect was observed with *IAR3-OE* or *ILR1-OE* (Fig. 4A, B). Lack of flower phenotype in *IAR3-OE* was not entirely unexpected given the relatively milder depletion of JA-Ile in *IAR3-OE* compared with *ILL6-OE* (Fig. 1; Supplementary Fig. S5).

In roots, both *ILL6-OE* and *IAR3-OE* exhibited resistance to exogenous JA-induced growth inhibition (Fig. 4C, D). Mutations in JA perception or signaling, defects in the conjugation step to produce JA-Ile (i.e. *jar1*), or increased turnover of JA-Ile (e.g. *CYP94B3* overexpression) can make plants resistant to exogenous JA (Lorenzo *et al.*, 2004; Staswick and Tiryaki, 2004; Thines *et al.*, 2007; Koo *et al.*, 2011). In *ILL6-OE* and *IAR3-OE*, increased hydrolysis of JA-Ile is likely to have made plants appear insensitive to exogenous JA. *IAR3-OE* and *ILL6-OE* roots were fully sensitive to exogenous coronatine, a structural mimic of JA-Ile and a potent agonist of the COI1–JAZ receptor system, indicating that the observed insensitivity to exogenous JA was not due to any defect in JA perception or signaling (Fig. 4E).

### Overexpression of ILR1 and IAR3 confers hypersensitivity to exogenous IAA conjugates whereas ILL6 antagonizes the process

**Fig. 5.**
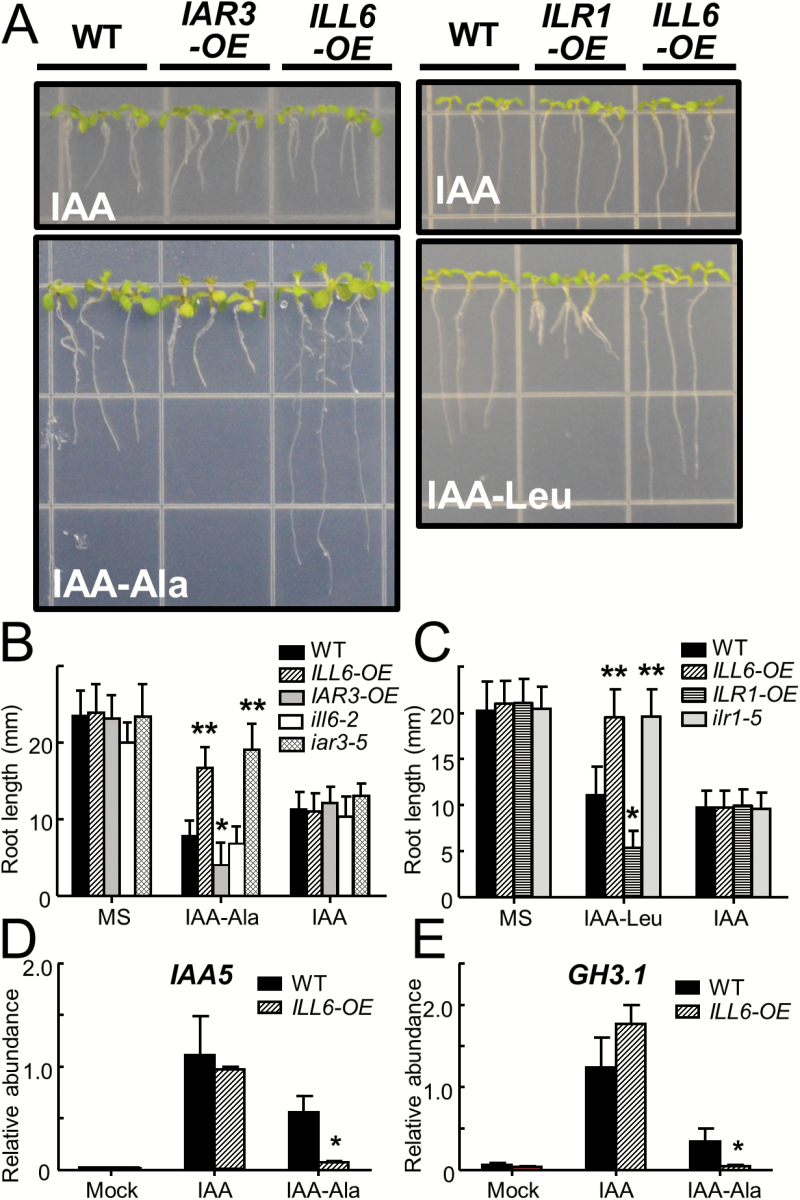
Impacts of genetic manipulation of IAHs on plant sensitivity to exogenous IAA conjugates. (A–C) Photographs and root length measurements of 9-day-old WT, *ILL6-OE* (line 5), *IAR3-OE* (line 42), *ILR1-OE*, *ill6-2*, *iar3-5*, and *ilr1-5* plants grown on MS medium supplemented or not with 5 μM IAA, 50 μM IAA-Ala, or 30 μM IAA-Leu. Data show the mean and SD (*n* >18). (D and E) qRT–PCR analysis of *IAA5* and *GH3.1* expression in leaves of 4-week-old WT and *ILL6-OE* plants sprayed (2h) with the mock, 5 μM IAA, or 50 μM IAA-Ala solutions. Relative abundance compared with *ACTIN8* is displayed. Error bars indicate the SD of three biological replicates. Asterisks denote a significant difference compared with the WT at *P*<0.05 (*) or *P*<0.001 (**); Student’s *t*-test. (This figure is available in colour at *JXB* online.)

An auxin-induced primary root growth inhibition assay was employed to examine auxin-related phenotypes. *ILL6-OE*, *IAR3-OE*, *ill6-2*, and *iar3-5* plants were grown together with WT control on MS medium supplemented or not with IAA (5 μM) or IAA-Ala (50 μM) (Fig. 5A, B). All genotypes grown on plain MS medium had similar root lengths, but their root growth was uniformly inhibited by IAA inclusion in the medium, indicating fully functional IAA perception and signaling pathways in all genotypes. However, when seedlings were grown on IAA-Ala, clearly visible variations in root lengths between genotypes appeared. WT root growth was inhibited by IAA-Ala. This could mostly be prevented by knocking-out IAR3 (*iar3-5*) and enhanced by overexpressing IAR3, consistent with IAR3’s dominant function in plant sensitivity to IAA-Ala (Davies *et al.*, 1999). The results were similar with *ilr1-5* and *ILR1-OE* on IAA-Leu plates (Fig. 5C), demonstrating the dominant function of ILR1 in the plant response to IAA-Leu (Bartel and Fink, 1995). Surprisingly, *ILL6-OE* roots were insensitive to IAA-Ala, reaching the same length as *iar3-5* roots (Fig. 5B). This was unexpected because ILL6 lacked any substrate specificity for IAA-Ala, and, even if it did have, it should make plants more sensitive to IAA-Ala, not insensitive (Table 1). Furthermore, the insensitivity of *ILL6-OE* to exogenous auxin conjugates was also apparent with IAA-Leu, with their roots reaching the length of the IAA-Leu-insensitive *ilr1-5* roots (Fig. 5C).

We tested whether the *ILL6-OE*-conferred resistance to IAA conjugates is observed in leaves. For this, expression of IAA response marker genes, *IAA5* and *GH3.1*, was measured 2h after spraying the mature rosette leaves of WT and *ILL6-OE* plants with solutions containing the mock treatment, IAA (5 μM), or IAA-Ala (50 μM). Both *IAA5* and *GH3.1* transcripts in the WT increased significantly upon IAA treatment and, to a lesser extent, upon IAA-Ala treatment compared with the mock treatment (Fig. 5D, E). In *ILL6-OE*, expression of the two genes was also induced by IAA, but not by IAA-Ala, reproducing a loss of responsiveness to IAA-Ala similar to that seen in the roots. The mechanism whereby ILL6 interferes with plant perception of IAA conjugates in *ILL6-OE* is unclear, but it is does not occur through transcriptional down-regulation of *IAR3* or *ILR1* genes because the expression of *IAR3* and *ILR1* genes was not impaired in the *ILL6-OE* leaves (Supplementary Fig. S9A–C at *JXB* online). In addition, we obtained indirect evidence that ILL6 could bind to IAA conjugates. As noted earlier, when GST–ILL6 is incubated with JA-Ile in *in vitro* hydrolysis assays, it hydrolyzes JA-Ile. However, when IAA-Ala was added along with JA-Ile, the JA-Ile hydrolysis by GST–ILL6 was strongly inhibited (Supplementary Fig. S9D), indicating that IAA-Ala was somehow interfering with GST–ILL6’s activity against JA-Ile, most probably through competition for the active site. It is possible then, that, in *ILL6-OE* plants, the highly abundant ILL6 proteins are competing with the relatively small number of endogenous IAR3 or ILR1 proteins for IAA conjugate substrates, preventing their hydrolysis by IAR3 or ILR1. In this case, exceeding concentrations of IAA conjugates in the root assay medium may eventually saturate the ILL6-binding sites and restore the root sensitivity to IAA conjugates. Supporting this hypothesis, a dose-dependent inhibition of root growth was observed with the *ILL6-OE* seedlings grown on increasing concentrations of IAA-Ala (Supplementary Fig. S9E).

### ILL6 and IAR3 are localized to the ER

**Fig. 6.**
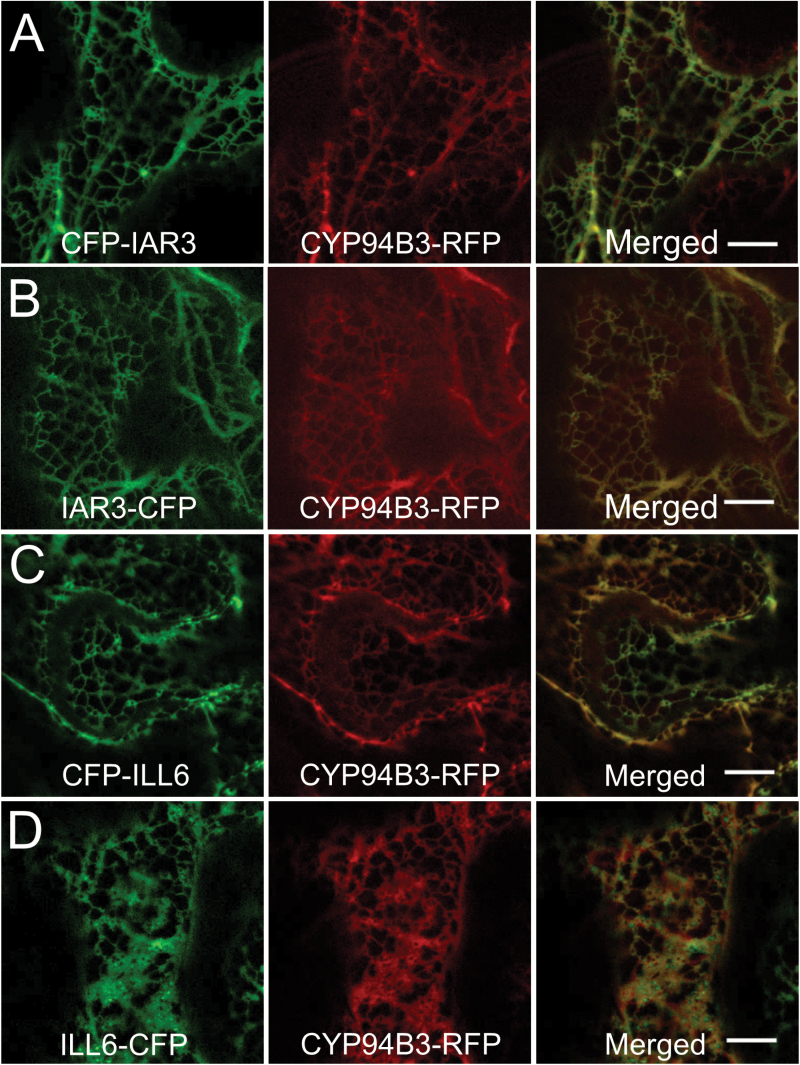
Subcellular localization of IAR3 and ILL6. Confocal images of tobacco leaf epidermal cells co-expressing CFP-fused IAR3 or ILL6 (left panels of A–D) with CYP94B3–mRFP (middle panels). CFP was fused to either the N- or C-terminus of IAR3 and ILL6, and designated as CFP–IAR3, IAR3–CFP, CFP–ILL6, and ILL6–CFP. Merges show co-localization (right panels of A–D). Scale bars=10 μm.

The ER has been the predicted site of subcellular location for several IAHs based on their primary sequence features (Ludwig-Müller, 2011). IAR3 has both the putative N-terminal signal sequence and the C-terminal ‘KDEL’ ER retrieval signature. ILL6, on the other hand, lacks the C-terminal ER retrieval motif but has the predicted N-terminal cleavable signal sequence. To determine experimentally the subcellular localization of IAR3 and ILL6 proteins, vector constructs with CFP-fused IAR3 and ILL6 were generated. Two versions of the constructs were tested for each protein where CFP was joined to either the N- or C-terminus of each protein, designated as CFP–IAR3, IAR3–CFP, CFP–ILL6, and ILL6–CFP. For N-terminal fusions, CFP was inserted behind the predicted signal sequence of IAR3 and ILL6, and, for the C-terminal fusions, CFP was inserted in front of the ‘KDEL’ of IAR3, whereas CFP was joined to the very end of the ILL6 protein. The resulting four constructs were transiently expressed in *N. benthamiana* leaves by syringe infiltration of *Agrobacterium* harboring each construct. A second culture of *Agrobacterium* carrying the CYP94B3–mRFP (monomeric red fluorescent protein) construct was co-infiltrated to be used as an ER marker (Koo *et al.*, 2014). Figure 6 displays laser scanning confocal microscopy images taken after 2 d of infiltration. All four CFP-fused IAR3 and ILL6 proteins illuminated the characteristic ER network which perfectly overlapped with the CYP94B3–mRFP signals. Thus, we conclude that both IAR3 and ILL6 proteins are targeted to the ER, making the ER the probable site of IAA and JA conjugate hydrolysis.

## Discussion

**Fig. 7.**
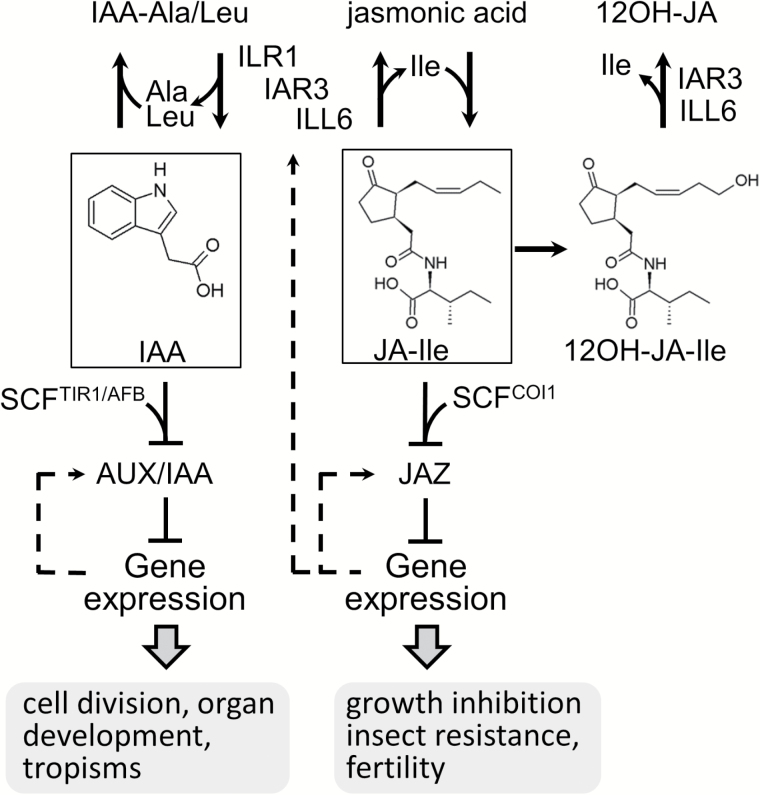
IAA and JA conjugation and deconjugation have opposite signaling consequences. IAA-Ala and IAA-Leu are hydrolyzed by IAR3 and ILR1, respectively, and the released free IAA activates IAA-responsive gene expression through the AUX/IAA–SCF^TIR1/AFB^ system. JA-Ile and 12OH-JA-Ile are hydrolyzed by either IAR3 or ILL6. As a result, JA-Ile-responsive gene expression through the JAZ-SCF^COI1^ system is attenuated. Genes encoding IAHs, AUX/IAA, and JAZ are under positive feedback regulation (dashed lines) along with other biosynthetic enzymes and transcriptional regulators.

Our results show that IAH enzymes contribute to the homeostasis of both auxin and JA in plants, providing a potential mechanism to regulate simultaneously the two hormone levels, which bear opposite signaling consequences of switching one on while switching off the other (Fig. 7).

Expression of the three genes was up-regulated by JA, but not by IAA, meaning that they are more likely to function under physiological conditions that induce JA biosynthesis. Mechanical tissue damage and insect herbivory are known to induce the otherwise low levels of JA in leaves dramatically. The function of ILL6 and IAR3 in these circumstances is probably to hydrolyze excess JA-Ile and thereby attenuate prolonged activation of stress responses. The potentially harmful effects of JA-Ile overaccumulation, however, were not apparent in *ill6iar3.* This is probably because JA-Ile eventually gets turned over via other pathway(s), such as the CYP94-mediated ω-oxidation pathway (Kitaoka *et al.*, 2011; Koo *et al.*, 2011; Heitz *et al.*, 2012). Higher order mutants that further block JA-Ile turnover will be useful to study the physiological effects of unrestrained JA-Ile accumulation.

Regarding the physiological impacts of IAA conjugate hydrolysis by IAHs, IAA conjugate content in mature Arabidopsis leaves is quite low (Kowalczyk and Sandberg, 2001) and is expected to be a relatively minor source for bulk free IAA in leaves. However, IAA conjugate hydrolysis may become more important for localized IAA increase in specific cell types (e.g. in undamaged meristematic regions of plants) where they could contribute to post-stress emergence of new organs. In addition to wounding or insect herbivory, IAHs may play a role based on a normal developmental program. Tissue-specific and developmentally controlled expression of *ILR1* and *IAR3* genes was studied using transgenic promoter–reporter lines of Arabidopsis (Rampey *et al.*, 2004). Both *ILR1* and *IAR3* were expressed in flowers, particularly in pollen, where interplay between auxin and JA signaling is required for fertility (Nagpal *et al.*, 2005). *IAR3* is also expressed in roots where collaborative actions of auxin and JA repress root meristem activity and growth (Chen *et al.*, 2011). Indeed, the root was where the strongest morphological effects of IAR3 and ILL6 overexpression were observed. Tissue-specific expression of *ILL6* has not been studied in detail; however, an available public microarray resource (http://bar.utoronto.ca/) indicates its expression in flowers, which coincides with reduced fertility of *ILL6-OE* plants. The remaining three members of IAHs (especially ILL2 which has high sequence homology to IAR3), even though they are not inducible by JA or wounding, could also potentially function as JA amidohydrolases in tissues where they are expressed. Catalytic activities of these other IAHs on JA conjugates remain to be tested.

A second aspect of auxin and JA pathway crosstalk mediated by IAHs is through overlapping substrate specificities of IAHs for both auxin– amino acid and JA–amino acid conjugates. Cross-regulation between different hormone signaling pathways by means of transcriptional regulation is common, but examples of crosstalk at the metabolic level are relatively rare. Purified recombinant GST–IAR3 enzyme showed the broadest substrate specificity among the three IAHs, being active against both IAA and JA conjugates, and, in addition, catalyzing the cleavage of all six amino acid-substituted conjugates of JA with similar efficiencies (Table 1). Somewhat differently from a previous report (Widemann *et al.*, 2013), hydroxylation at the C_12_ position of JA-Ile did not diminish the hydrolysis by GST–IAR3. On the other hand, further modification of the -OH group by oxidation (to form -COOH), glucosylation, or sulfation abolished their hydrolysis by GST–IAR3 (Table 1). The versatility of IAR3 is also shown by its ability to hydrolyze synthetic abscisic acid–amino acid conjugates *in vitro* (Todoroki *et al.*, 2011).

GST–ILR1 displayed more stringent substrate specificities compared with GST–IAR3, with the most notable difference being its inability to use JA-Ile, making it a more specific enzyme for auxin conjugates. GST–ILR1 showed appreciable activity toward conjugates other than JA-Ile (e.g. JA-Phe and JA-Leu), but these conjugates occur at much lower levels than JA-Ile in Arabidopsis seedlings and wounded leaves (Staswick and Tiryaki, 2004; Koo *et al.*, 2009). JA-Leu and JA-Phe were also ineffective in inhibiting root elongation (Staswick and Tiryaki, 2004) and were not effective as ligands to promote COI1 interaction with JAZs (Thines *et al.*, 2007). Consistently, knocking-out or overexpressing ILR1 had no measurable impact on the endogenous JA profile or visible JA-deficient phenotypes. However, we cannot rule out the possibility that ILR1’s function as a JA conjugate hydrolase could become important in certain conditions, cell types, or plants species, where these rarer JA conjugates accumulate to higher concentrations (Widemann *et al.*, 2015).

Both *in vitro* and *in vivo* results are consistent with ILL6 being a JA-specific amidohydrolase. Even though GST–ILL6’s *in vitro* hydrolytic activity for JA-Ile was an order of magnitude lower than that of GST–IAR3, overexpression of ILL6 in plants (*ILL6-OE*) resulted in a severe depletion of JA-Ile. This stark difference between *in vitro* and *in vivo* activities illustrates the importance of proper contextual requirements for optimal ILL6 activity whether that is protein folding, cofactors, or post-translational modification. Surprisingly, even though GST–ILL6 enzyme displayed no *in vitro* hydrolytic activity against IAA-Ala or IAA-Leu, overexpression of ILL6 made plants strongly insensitive to these IAA conjugates, suggesting the potential function of ILL6 as a negative regulator of IAA–amino acid hydrolysis *in vivo*. IAA perception and signaling in *ILL6-OE* was normal, and no sign of changes in *IAR3* or *ILR1* transcription was detected to explain *ILL6-OE*’s insensitivity to IAA conjugates. We found out that the JA-Ile-hydrolyzing activity of GST–ILL6 was strongly inhibited by the addition of IAA-Ala (a non-suitable substrate for ILL6) to the reaction mixture along with JA-Ile substrates (Supplementary Fig. S9D at *JXB* online). This suggests that even though ILL6 cannot hydrolyze IAA-Ala, it may still bind IAA-Ala, which, by an unknown mechanism, interferes with the JA-Ile hydrolysis by ILL6. It can be speculated that in the *ILL6-OE* seedlings, high levels of ILL6 proteins may compete with the less abundant native IAR3 or ILR1 proteins for IAA conjugate substrates, preventing their hydrolysis by IAR3 or ILR1, and thus making plants appear resistant to the IAA conjugates. Our data showing restoration of *ILL6-OE*’s sensitivity to the increasing concentrations of IAA-Ala are consistent with this hypothesis. More study is needed to investigate this novel function of ILL6 as a negative regulator of IAA conjugate hydrolysis.

12OH-JA that forms as a result of 12OH-JA-Ile hydrolysis can be considered an inactive by-product of the JA catabolic pathway; however, specific signaling roles for 12OH-JA have been reported in other plant species (Yoshihara *et al.*, 1989; Nakamura *et al.*, 2011; Patkar *et al.*, 2015). Presently, hydrolysis of 12OH-JA-Ile is the only experimentally proven metabolic pathway to produce 12OH-JA in higher plants (Fig. 7). However, the close to 50% of the WT level of residual 12OH-JA in the *iar3ill6* double mutant (Fig. 2) indicates the existence of separate metabolic routes to make 12OH-JA. Given the low expression level of the remaining members of *IAH* genes in wounded leaves and the lack of 12OH-JA-Ile hydrolytic activity by ILR1, a contribution of other members seems less likely. Recently, a monooxygenase from rice blast fungus was reported to catalyze direct 12-hydroxylation of JA (Patkar *et al.*, 2015), but such an enzyme has yet to be identified in plants.

One question regarding IAH’s contribution to the attenuation of JA signaling is: ‘What is the net effect of the JA-Ile hydrolysis to JA, which could potentially be conjugated back to JA-Ile by JAR1?’ We do not have direct evidence as to whether or how much of the hydrolyzed JA is recycled back to synthesize JA-Ile. However, the contribution by this recycling to the overall JA-Ile level is expected to be far less than JA-Ile hydrolysis, resulting in a net decrease of JA-Ile over time. This is partly because only a fraction of bulk JA, typically ~10%, is converted to JA-Ile in leaves. JA-Ile-deficient phenotypes of *IAR3-OE* and *ILL6-OE* plants are consistent with JA-Ile signal being destroyed through hydrolysis rather than being futile. Differential partitioning between subcellular compartments may also play a role in physical separation of the cleaved JA from JA-Ile. In contrast to the cytosolic location of the JA-Ile-conjugating enzyme, JAR1 (Hsieh *et al.*, 2000), we have provided evidence that the hydrolases are localized to the ER, together with the CYP94 enzymes catalyzing the oxidative turnover of JA-Ile (Koo *et al.*, 2014). The close proximity of the hydrolases to the hydroxylases may facilitate their collaborative turnover of JA-Ile. The exact topology of these proteins remains to be determined. Based on the established topology of many ER-localized P450s, CYP94s were assumed to face the cytosolic face of the ER (Koo *et al.*, 2014). However, IAR3 which has both an N-terminal signal sequence and a C-terminal ‘KDEL’ ER retrieval signature may have different topology from CYP94s or ILL6 which lacks the C-terminal ER retrieval motif. Strong resistance to exogenous JA displayed by the overexpressing plants suggests effective competition by the ER-residing hydrolases with the CYP94s and the nuclear-residing receptor complexes for the JA-Ile substrate. This indicates rapid interchange of JA-Ile between different subcellular compartments where these enzymes can have access to it. The situation may be analogous to the IAA conjugates, where ER-localized IAHs are hydrolyzing and activating the IAA conjugates made by the cytosolic GH3 enzymes (Wright *et al.*, 1987; Ludwig-Müller, 2011).

Additional studies are needed to understand the biological function of IAA and JA conjugate hydrolysis and the advantage provided by the simultaneous regulation of auxin and JA levels by IAHs. This may be too subtle to be detected easily. However, strong evolutionary conservation across higher plants of the amidosynthetase and amidohydrolase family genes as well as the ubiquitous occurrence of amino acid conjugates of auxin and JA (Ludwig-Müller, 2011; Wasternack and Hause, 2013) bear witness to their importance for plant survival in nature.

## Supplementary data

Supplementary data are available at *JXB* online.

Table S1. *ILL6-OE* plants have reduced fertility.

Table S2. Primers used in this study.

Figure S1. Arabidopsis *IAH* family gene expression.

Figure S2. *In vitro* hydrolysis activities of GST–IAR3, GST–ILL6, and GST–ILR1.

Figure S3. Generation of *ILL6-OE* plants.

Figure S4. Generation of *IAR3-OE* plants.

Figure S5. JA-Ile in the homozygous T_3_ *ILL6-OE* and *IAR3-OE* plants.

Figure S6. Jasmonic acid and 12COOH-JA-Ile accumulation in IAH-overexpressing lines.

Figure S7. Molecular characterization of *iar3-5ill6-2*.

Figure S8. *JAR1* and *GH3.1* gene expression in *IAR3-OE* and *ILL6-OE*.

Figure S9. Possible mechanism of *ILL6-OE*-conferred insensitivity to IAA conjugates.

Supplementary Data
