# Supplementary material for: Hormone crosstalk in wound stress response: wound-inducible amidohydrolases can simultaneously regulate jasmonate and auxin homeostasis in Arabidopsis thaliana
Source: J Exp Bot. 2015 Dec 15;67(7):2107–20. doi: 10.1093/jxb/erv521 (PMC4793799; doi:10.1093/jxb/erv521)
Supplement: Supplementary Data [file supp_67_7_2107__index.html]

Hormone crosstalk in wound stress response: wound-inducible amidohydrolases can simultaneously regulate jasmonate and auxin homeostasis in Arabidopsis thaliana — Hormone crosstalk in wound stress response: wound-inducible amidohydrolases can simultaneously regulate jasmonate and auxin homeostasis in Arabidopsis thaliana — Supplementary Data 

# Hormone crosstalk in wound stress response: wound-inducible amidohydrolases can simultaneously regulate jasmonate and auxin homeostasis in *Arabidopsis thaliana*

## Supplementary Data

Data files

- supplementary\_tables\_S1\_S2\_figures\_S1\_S9.pdf - Supplementary Data
