# Supplementary material for: Hormone crosstalk in wound stress response: wound-inducible amidohydrolases can simultaneously regulate jasmonate and auxin homeostasis in Arabidopsis thaliana
Source: J Exp Bot. 2015 Dec 15;67(7):2107–20. doi: 10.1093/jxb/erv521 (PMC4793799; doi:10.1093/jxb/erv521)
Supplement: Supplementary Data [file supp_erv521_supplementary_tables_S1_S2_figures_S1_S9.pdf]

Title:

Hormone crosstalk in wound stress response: Wound-inducible amidohydrolases can simultaneously regulate jasmonate and auxin homeostasis in *Arabidopsis thalinana*.

Authors:

Tong Zhang, Arati N. Poudel, Jeremy B. Jewell, Naoki Kitaoka, Paul Staswick, Hideyuki Matsuura, and Abraham J. Koo

Supplementary Tables

Table S1. *ILL6-OE* plants have reduced fertility.

Line*	Under-developed siliques†	Normal siliques	Total siliques counted	% under-developed siliques
WT	2	88	90	2
ILL6-OE #5	**57**	**47**	**104**	**55**
ILL6-OE #19	6	90	96	6
ILL6-OE #47	**53**	**34**	**87**	**61**
ILL6-OE #63	11	78	89	12

* Four representative T2 plants were scored for silique development. Bold indicates lines exhibiting reduced fertility.

† Siliques < 4 mm were counted as underdeveloped.

Supplementary Table S2. Primers used in this study.

Use		Primer name	Primer sequences (5'–3')
T-DNA genotyping			
	T-DNA	LBb1	GCGTGGACCGCTTGCTGCAACT
	ill6-2	Salk024894.LP	GACTATGCTTCTTGGTGCTGC
		Salk024894.RP	CGCACCTCTTGAATACGTTTC
	iar3-5	iar3-5 LP	GTTCTCCACGTGCGTTATAGC
		iar3-5 RP	AAAAAGCCACACTGTTCCATG
Transgenic plants			
	ILL6OE	ILL6_XbaI F	GCTCTAGAATGGACAATCTCCGGAAAC
		ILL6_XhoI R	GCCTCGAGTTATGAATGTTTATCATTTAAGTATCTC
	IAR3OE	IAR3_XbaI F	GTCTAGAATGAGTTTCTTCAAATGGG
		IAR3_BamHI R	CGGATCCTCAAAGTTCATCTTTTTTGTTACTC
Recombinant proteins			
	GST-ILL6	ILL6_EcoRI F	GAATTCACCAACTTACCTTTCTTTGAAGTG
		ILL6_SalI R	GTCGACTTATGAATGTTTATCATTTAAG
	GST-IAR3	IAR3_EcoRI F	GAATTCTCCTCTAATGGGTTATCTCAAATAC
		IAR3_SalI R	GTCGACTCAAAGTTCATCTTTTTTGTTACTCT
Subcellular localization			
	CFP-ILL6	gw_ILL6_sp F	CACCATGGACAATCTCCGGAAAC
		ILL6 R	TTATGAATGTTTATCATTTAAGTATCTCTCAGC
		ov_ILL6sp-CFP F	GTCTCTCTTACCATAGCCGTGAGCAAGGGCGAG
		ov_ILL6sp-CFP R	CTCGCCCTTGCTCACGGCTATGGTAAGAGAGAC
		ov_CFP-ILL6(noSP) F	GACGAGCTGTACAAGACCAACTTACCTTTCTTTG
		ov_CFP-ILL6(noSP) R	CAAAGAAAGGTAAGTTGGTCTTGTACAGCTCGTC
	CFP-IAR3	gw_IAR3_sp F	CACCATGAGTTTCTTCAAATGGGTTTC
		IAR3 R	TCAAAGTTCATCTTTTTTGTTACTCTTATTTAGAG
		ov_IAR3sp-CFP F	CCGACTCTGATTTCATGTGTGAGCAAGGGCGAG
		ov_IAR3sp-CFP R	CTCGCCCTTGCTCACACATGAAATCAGAGTCGG
		ov_CFP-IAR3(noSP) F	GACGAGCTGTACAAGTCCTCTAATGGGTTATCT
		ov_CFP-IAR3(noSP) R	AGATAACCCATTAGAGGACTTGTACAGCTCGTC
	ILL6-CFP	CFP R	TCACTTGTACAGCTCGTCCATG
		ov_ILL6-CFP F	GATACTTAAATGATAAACATTCAGTGAGCAAGGGCGAG
		ov_ILL6-CFP R	CTCGCCCTTGCTCACTGAATGTTTATCATTTAAGTATC

Supplementary Table S2. Continued.

Use	Primer name	Primer sequences (5'–3')
Subcellular localization		
IAR3-CFP	CFP_KDEL R	TCAAAGTTCATCTTTCTTGTACAGCTCGTCCATG
	ov_IAR3-CFP F	CAACTCTAAATAAGAGTAACAAAGTGAGCAAGGGCGAG
	ov_IAR3-CFP R	CTCGCCCTTGCTCACTTTGTTACTCTTATTTAGAGTTG
qRT-PCR and RT-PCR		
ACTIN8	rt ACTIN8 F	GAGACAACTTACAACTCGATC
	rt ACTIN8 R	CTGTGGACAATGCCTGGAC
ILL1	rt ILL1 F	CTGTCAACCTTGGTACAGTTTCC
	rt ILL1 R	TTGTTCTACGAATTAAGGCTTTTCATC
ILL2	rt ILL2 F	ACCTTGGTTGTAGACAGGTTGTTC
	rt ILL2 R	TATAACACTTGCAAGGATCTTGTACG
ILL3	rt ILL3 F	GTGATTCTGAAGCAATATTTGGGATG
	rt ILL3 R	CTAAGATGGTAGATGATGCAGCC
ILL5	rt ILL5 F	CGCTTGCTATTATGTGAGTTCTTGG
	rt ILL5 R	GCAGAAACTCATCGTGAATCAAGAGA
ILL6	rt ILL6 F	GGTCCATGTGTCCCATATCC
	rt ILL6 R	AGCTTCACGGGATACAATGC
ILR1	rt ILR1 F	CTTAGATGGGATACTCAGTGTTC
	rt ILR1 R	CAATCTGTTGCAAGGCAACAAC
IAR3	rt IAR3 F	TCATCATCACGCACACCTCTCTTA
	rt IAR3 R	GAAACCCATTTGAAGAAACTCAT
GH3.1	rt GH3.1 F	CGATCGTCGCCAGCTTCTTTAC
	rt GH3.1 R	CCCGGCACATACAAATTCATTACG
IAA5	rt IAA5 F	TCCGCTCTGCAAATTCTGTTCG
	rt IAA5 R	ACGATCCAAGGAACATTTCCCAAG
JAZ8	rt JAZ8 F	ATGAAGCTACAGCAAAATTGTG
	rt JAZ8 R	GACCCGTTTGAGGATGACTTG
JAR1	rt JAR1 F	CTCACTGGTCACCCTGTTCCTG
	rt JAR1 R	GAAGGCAAAAGCAGTGCGAAACAG

Supplementary Figures

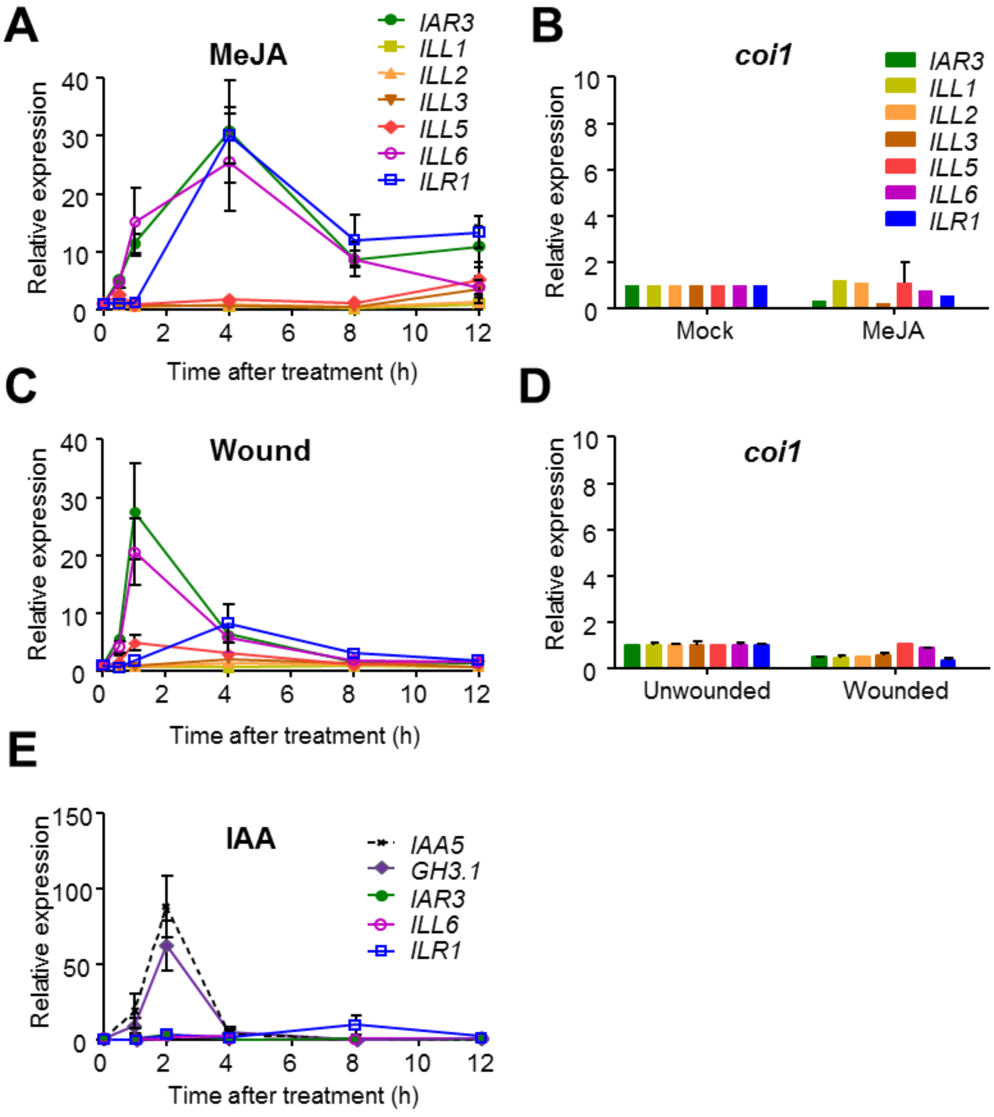

Fig. S1. (A-D) Arabidopsis *IAH* family gene expression in response to MeJA (A and B), wounding (C and D), or IAA (E) treatments in WT (A, C, E) or *coi1* (B and D) plants. Rosette leaves of four-week old plants were either mechanically wounded with a hemostat or sprayed with 100 μM MeJA or 5 μM IAA. Transcript abundance was determined by qRT-PCR. Relative expression levels were normalized by *ACTIN8* as internal reference gene and displayed as fold-change compared to the levels of untreated (time zero) WT. Each data point is the mean ± SD of three biological replicates.

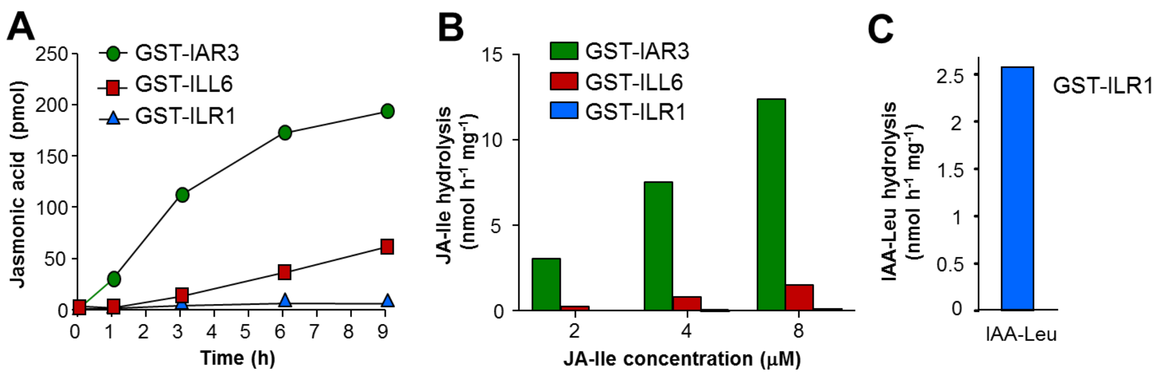

Fig. S2. *In vitro* hydrolysis activities of purified GST-IAR3, GST-ILL6, and GST-ILR1 expressed in *E. coli*. (A) Time course of JA-Ile cleavage. Five µg of purified proteins were incubated with 4 μM JA-Ile for indicated durations. Jasmonic acid release over time was quantified using UPLC-MS/MS. (B) Proteins were incubated with increasing concentrations of JA-Ile for 3 h before quantification of jasmonic acid. (C) GST-ILR1 was incubated with 4 μM IAA-Leu for 0.5 h followed by IAA quantification.

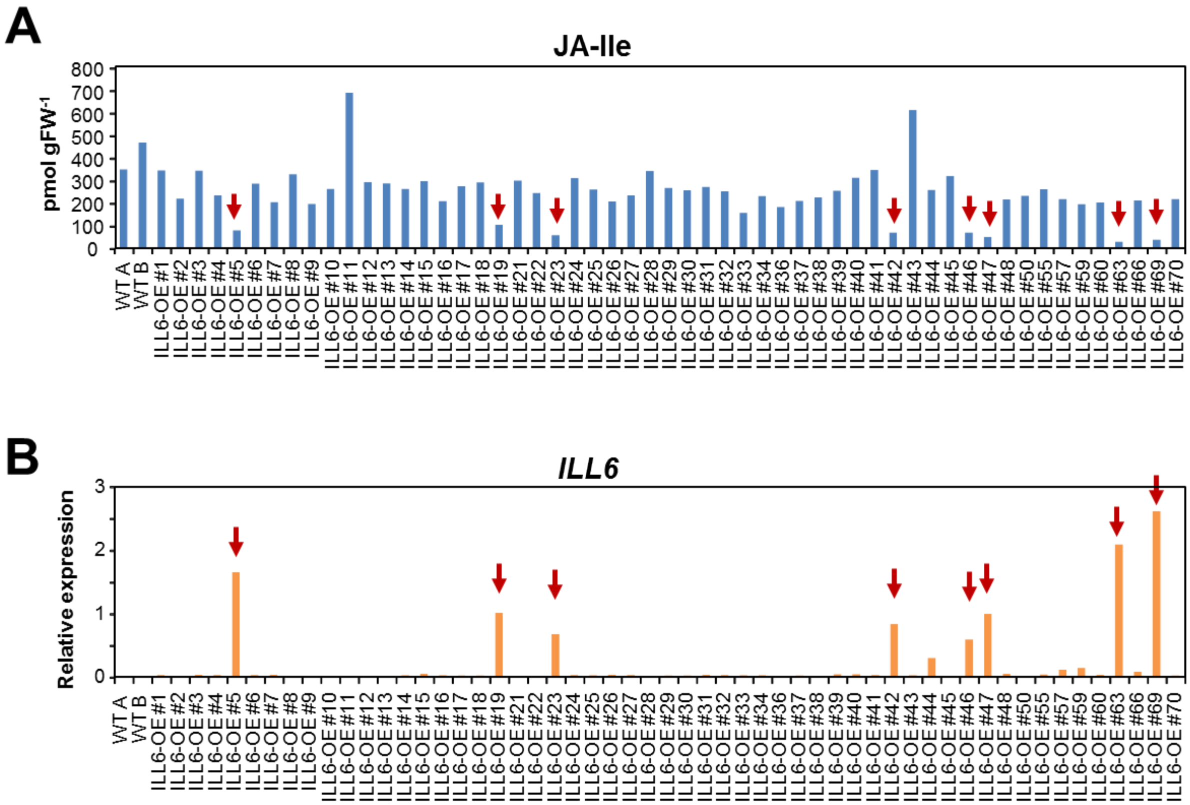

Fig. S3. Generation of transgenic Arabidopsis lines overexpressing *ILL6* (*ILL6-OE*). (A) JA-Ile levels in the wounded (2 h) leaves of WT and 55 independent kanamycin resistant *ILL6-OE* T1 lines. Endogenous JA-Ile levels were quantified using UPLC-MS/MS. (B) *ILL6* transcript levels in the leaves of WT and *ILL6-OE* lines. Leaf tissues for RNA analysis were collected from unwounded plants to measure the constitutive expression of *ILL6* transgene. *ILL6* transcript levels were determined by qRT-PCR and expressed relative to the level of *ACTIN8*. Red arrows indicate eight lines displaying strict correlation between the increased *ILL6* transcript level and decreased JA-Ile level.

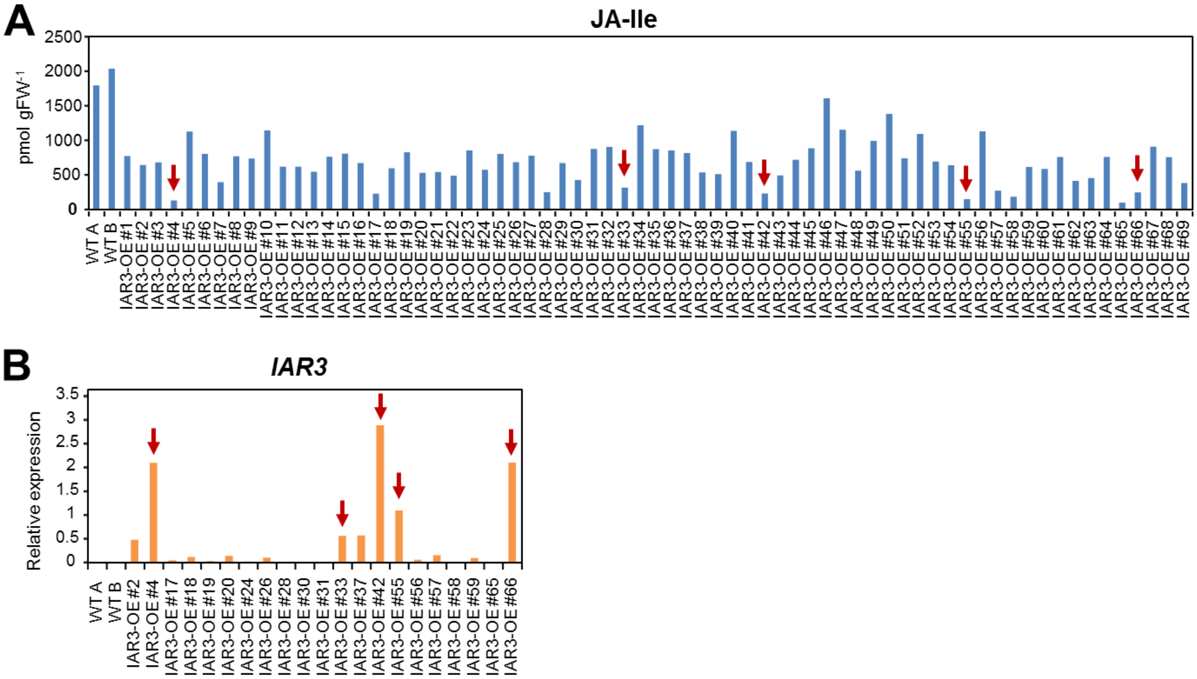

Fig. S4. Generation of transgenic Arabidopsis lines overexpressing *IAR3* (*IAR3-OE*). (A) JA-Ile levels in the wounded (2 h) leaves of WT and 69 independent kanamycin resistant *IAR3-OE* T1 lines. Endogenous JA-Ile levels were quantified using UPLC-MS/MS. (B) *IAR3* transcripts in the leaves of WT and 21 selected *IAR3-OE* lines.

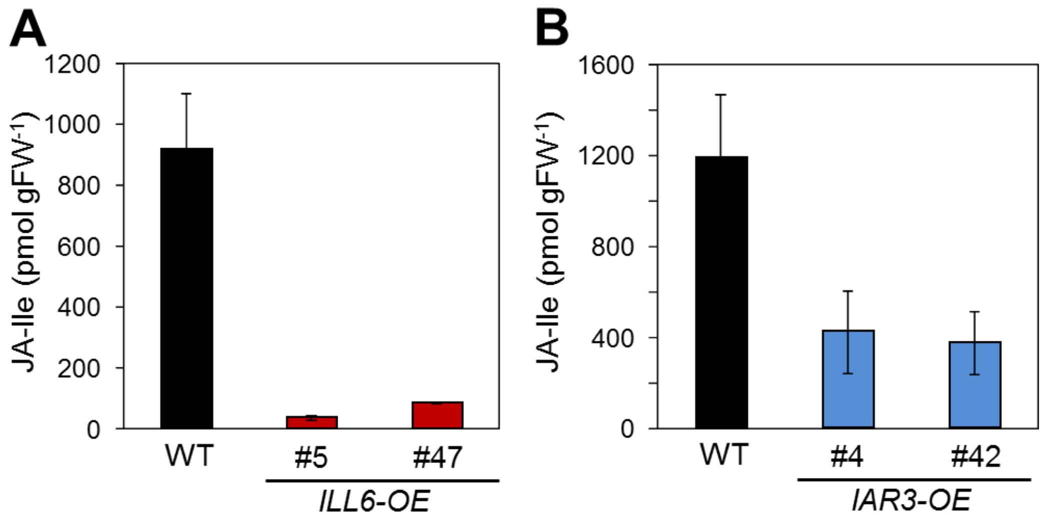

Fig. S5. JA-Ile is reduced in the homozygous T3 generation *ILL6-OE* (progenies of T1 lines 5 and 47) *IAR3-OE* (lines 4 and 42). JA-Ile levels were quantified after 2 h of wounding the leaves. Data show mean ± SD of 3 replicates.

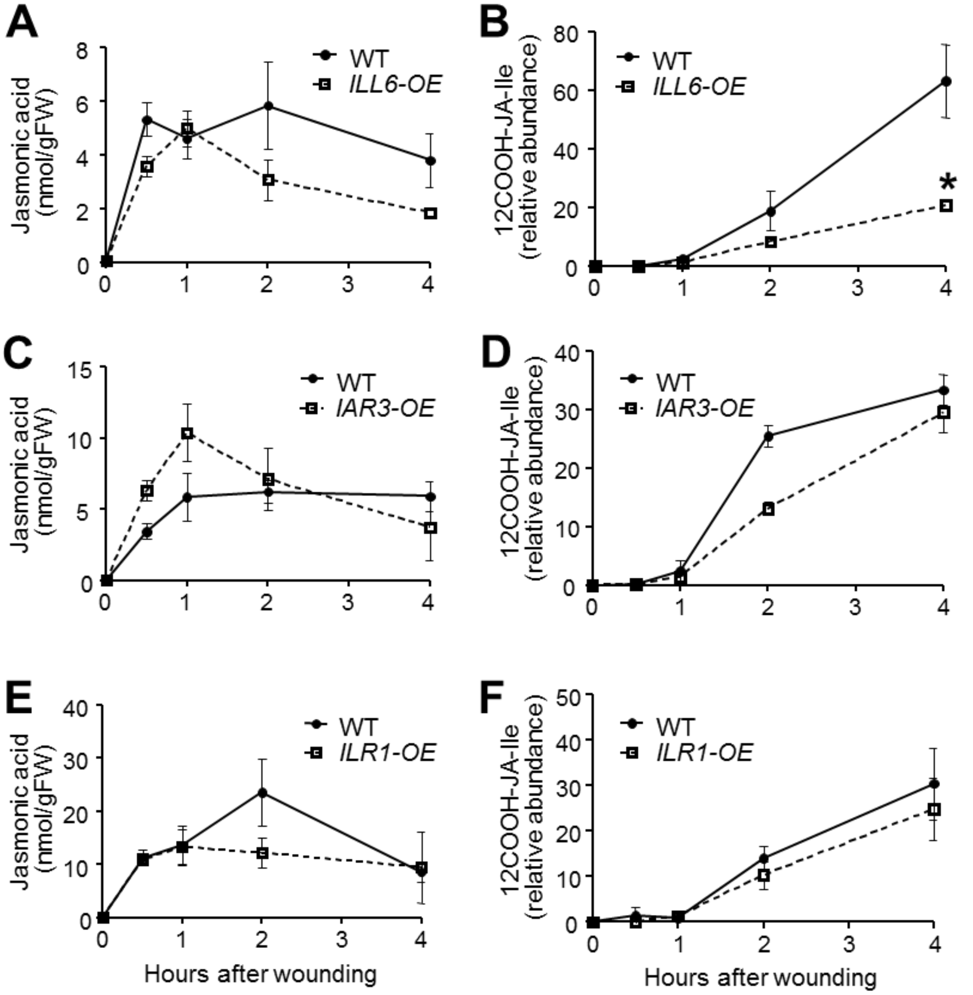

Fig. S6. Time course of jasmonic acid (A, C, E) and 12COOH-JA-Ile (B, D, F) accumulation in wounded leaves of WT, *ILL6-OE* (line 5), *IAR3-OE* (line 42), and *ILR1-OE* plants. Mechanically damaged leaves were harvested at 0, 0.5, 1, 2, 4 h after wounding for hormone quantification by UPLC-MS/MS. Each data point represents the mean ± SD of three biological replicates. Asterisks denote significant difference compared to WT at $p < 0.05$, Student's *t* test.

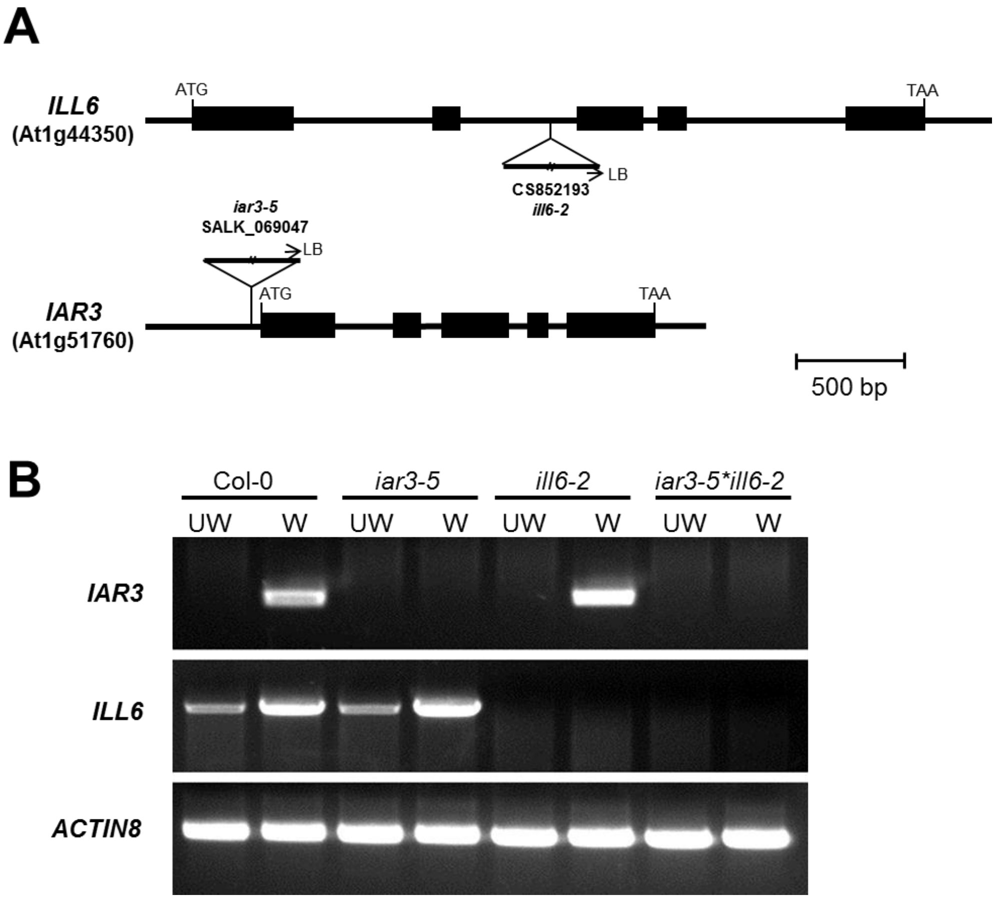

Fig. S7. Molecular characterization of the double homozygous *iar3-5ill6-2* mutant. (A) Schematics of the genomic structures of *ILL6* and *IAR3* genes and the positions of T-DNA insertions. Exons are indicated by black rectangles and arrows show direction of the left border (LB) primers used for PCR genotyping. (B) RT-PCR analyses showing the absence of *IAR3* and *ILL6* transcripts in wounded (W) and unwounded (UW) leaves of the indicated single (*iar3-5* or *ill6-2*) and double insertion lines (*iar3-5ill6-2*). Primers used for genotyping or RT-PCR are shown in Supplementary Table S2.

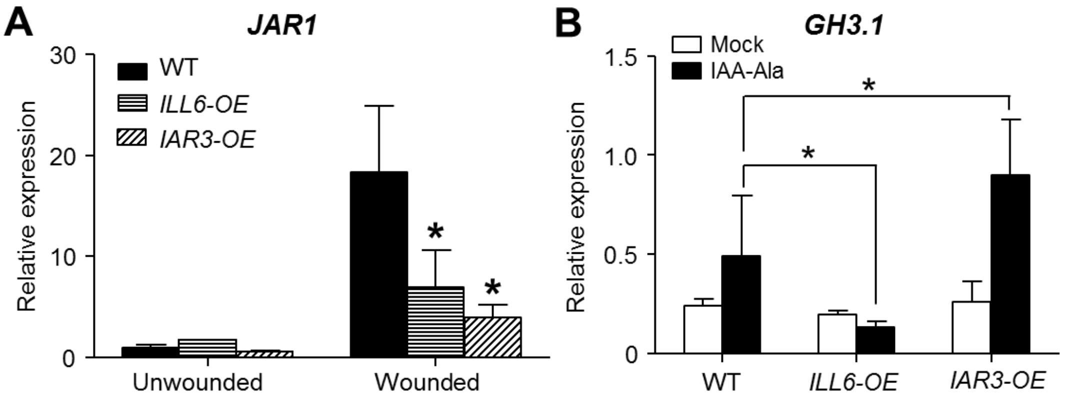

Fig. S8. Overexpression of IAR3 and ILL6 impacts JA and IAA marker gene expression. (A) qRT-PCR analysis of *JAR1* expression in unwounded and wounded (2 h) leaves of WT, *ILL6-OE* (line 5), and *IAR3-OE* (line 42). Fold change relative to unwounded WT transcript level is displayed. (B) *GH3.1* expression in 9-d old seedlings grown on MS media containing the mock or 50 μM IAA-Ala. Expression levels relative to *ACTIN8* are displayed. Error bars denote SD of three biological replicates. Asterisks indicate significant difference at $p < 0.05$ compared to WT, Student's *t* test.

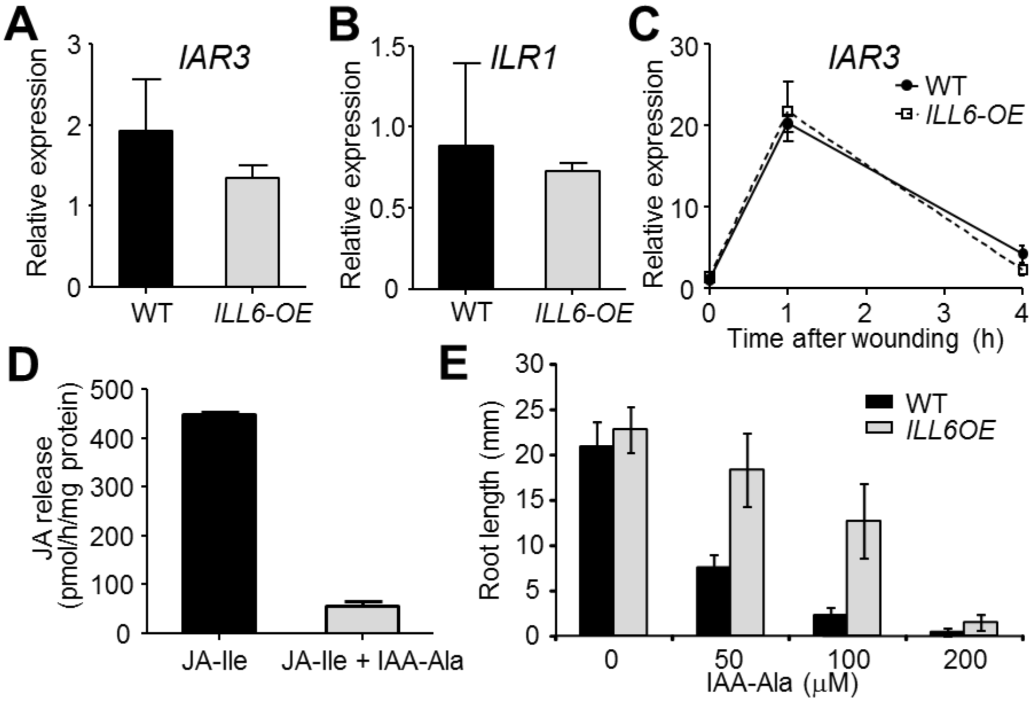

Fig. S9. Possible mechanism of *ILL6-OE*-conferred insensitivity to IAA conjugates. (A and B) *IAR3* and *ILR1* transcript levels in mature rosette leaves of WT and *ILL6-OE* sprayed (2 h) with 50 μM IAA-Ala. (C) Time course of *IAR3* expression in wounded leaves of four-week old WT and *ILL6-OE*. Transcript levels were determined by qRT-PCR and were expressed either as relative values to *ACTIN8* (A and B) or as fold-induction compared to the unwounded WT (0 h) (C). Data show mean ± SD of three biological replicates. (D) JA-Ile hydrolysis activity of GST-ILL6 is inhibited by the presence of IAA-Ala. *In vitro* hydrolysis assay was conducted on purified GST-ILL6 proteins using JA-Ile (4 μM) as substrate in the presence and absence of equal concentration of IAA-Ala (4 μM). Data show mean and SD of three biological replicates. (E) Root length assay of nine-day old WT and *ILL6-OE* grown on MS media supplemented with 0, 50, 100, and 200 μM IAA-Ala. Data show the mean ± SD (n > 14).
